# Modulation of Structure and Dynamics of Cardiac Troponin by Phosphorylation and Mutations Revealed by Molecular Dynamics Simulations

**DOI:** 10.1021/acs.jpcb.3c02337

**Published:** 2023-10-04

**Authors:** Zeyu Yang, Steven B. Marston, Ian R. Gould

**Affiliations:** † Department of Chemistry, Molecular Sciences Research Hub, Imperial College London, Shepherd’s Bush, London W12 0BZ, U.K.; ‡ Institute of Chemical Biology, Molecular Sciences Research Hub, Imperial College London, Shepherd’s Bush, London W12 0BZ, U.K.; § National Heart & Lung Institute, Imperial College London, London W12 0NN, U.K.

## Abstract

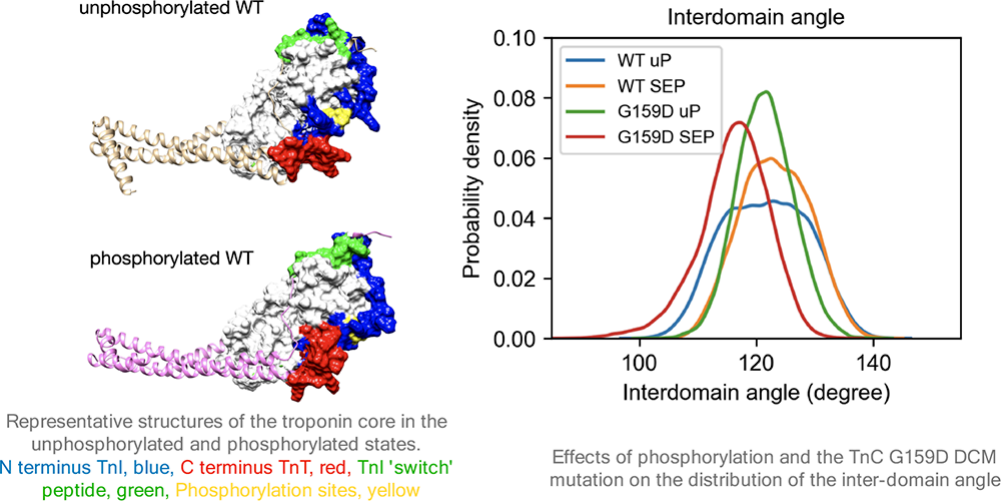

Adrenaline acts on β1 receptors in the heart muscle to enhance contractility, increase the heart rate, and increase the rate of relaxation (lusitropy) via activation of the cyclic AMP-dependent protein kinase, PKA. Phosphorylation of serines 22 and 23 in the N-terminal peptide of cardiac troponin I is responsible for lusitropy. Mutations associated with cardiomyopathy suppress the phosphorylation-dependent change. Key parts of troponin responsible for this modulatory system are disordered and cannot be resolved by conventional structural approaches. We performed all-atom molecular dynamics simulations (5 × 1.5 μs runs) of the troponin core (419 amino acids) in the presence of Ca^2+^ in the bisphosphorylated and unphosphorylated states for both wild-type troponin and the troponin C (cTnC) G159D mutant. PKA phosphorylation affects troponin dynamics. There is significant rigidification of the structure involving rearrangement of the cTnI(1–33)–cTnC interaction and changes in the distribution of the cTnC helix A/B angle, troponin I (cTnI) switch peptide (149–164) docking, and the angle between the regulatory head and ITC arm domains. The familial dilated cardiomyopathy cTnC G159D mutation whose Ca^2+^ sensitivity is not modulated by cTnI phosphorylation exhibits a structure inherently more rigid than the wild type, with phosphorylation reversing the direction of all metrics relative to the wild type.

## Introduction

Interaction between myosin filaments and actin-based thin filaments in cardiac muscle generates a contractile force that causes the propulsion of blood around the circulatory system. Ca^2+^ acts on troponin and tropomyosin to switch the thin filament on and off; however, in cardiac muscle, a more graded form of regulation is needed to tailor cardiac output to the body’s needs. This is achieved by the action of adrenaline on β1 receptors in heart muscle cells, leading to enhanced contractility and a faster heart rate. β1 receptor activation leads to cAMP production. cAMP acts directly on membrane channels and activates the cyclic AMP-dependent protein kinase, PKA. PKA phosphorylates a number of membrane and contractile proteins including two serines (22 and 23) located in a 33-amino-acid N-terminal cardiac-specific extension to cardiac troponin I (cTnI).^1,2^ Phosphorylation of serines 22 and 23 causes a two- to threefold decrease in myofilament Ca^2+^ sensitivity and a corresponding increase in the Ca^2+^ dissociation rate that determines the relaxation rate.^3,4^ This small change is sufficient to allow the lusitropic response to adrenergic stimulation, since the faster relaxation rate of the cardiac muscle due to cTnI phosphorylation contributes to shortening the cardiac muscle contraction–relaxation cycle, allowing for efficient contraction at a faster heart rate.

**Figure 1:**
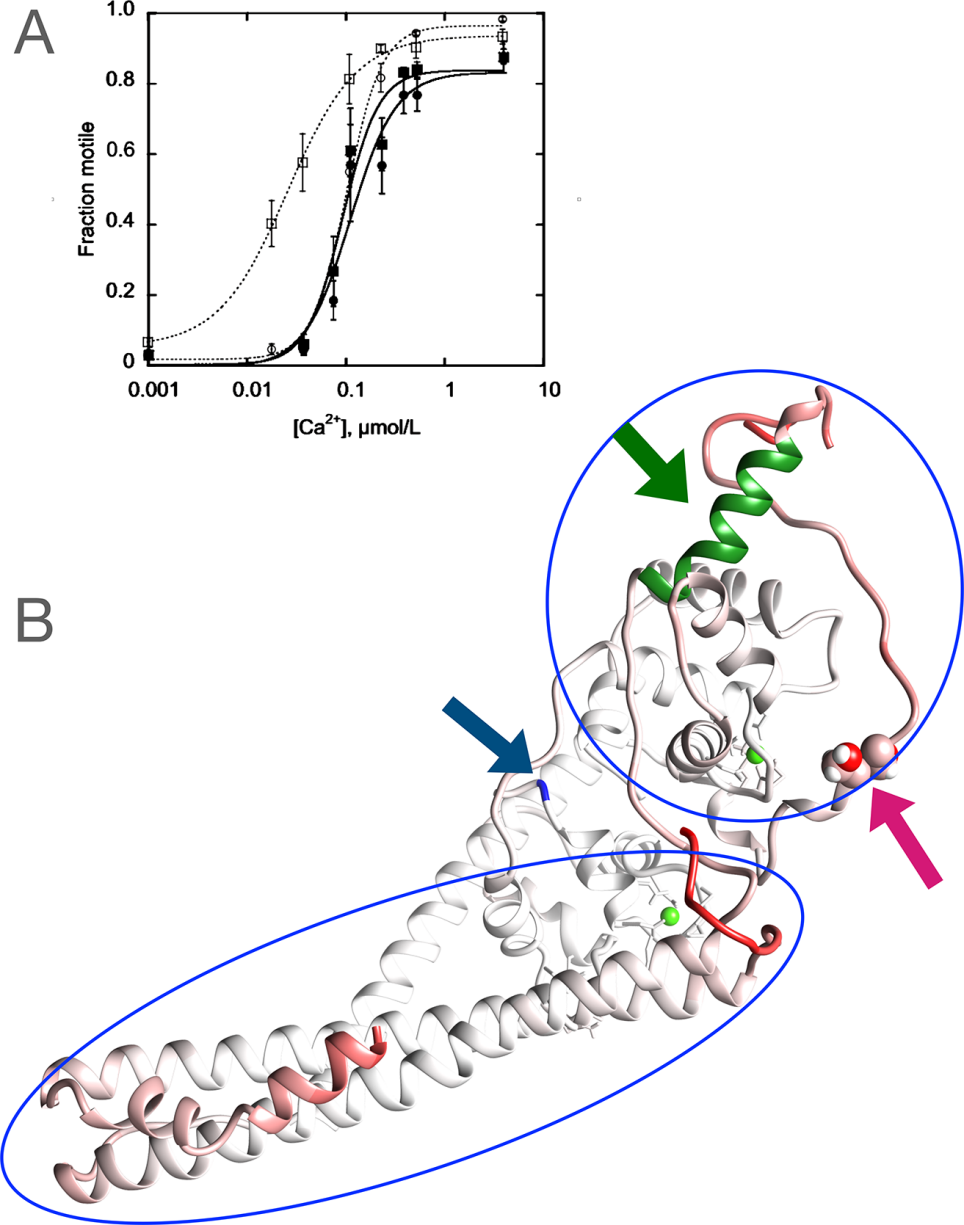
(A) The effects of phosphorylation and the G159D mutation on Ca^2+^activation of thin filaments. The plots show the decrease in Ca^2+^ sensitivity upon phosphorylation and the absence of this shift (uncoupling) due to the G159D mutation, measured by an *in vitro* motility assay. Dotted lines, open points wild type; solid lines and points, G159D; unphosphorylated TnI, squares; phosphorylated TnI, circles. Mean and SEM are shown. The lines are fits of the data to the Hill equation. Replotted from data of Dyer et al.^7^ (B) Representative structure of the backbone of the unphosphorylated troponin core from molecular dynamics simulation. The residues are colored according to their root mean square fluctuation (RMSF), with the highest RMSF as the deepest red. The locations of the phosphorylatable serines, 22 and 23, in NcTnI (magenta arrow), cTnC G159 (blue arrow), and the switch peptide (green arrow) are indicated. Bound Ca^2+^ ions are colored in green. The N-terminal domains of troponin C (NcTnC) and of the ITC domain are outlined in blue. The ITC domain contains the C-terminus of TnC (94–161), helices H1 and H2 of cTnI (42–136), and helices H1 and H2 of cTnT (201–277).

Many mutations in thin-filament proteins have been found to be associated with inherited heart disease, notably hypertrophic cardiomyopathy (HCM) and familial dilated cardiomyopathy (DCM). Importantly, such mutations commonly affect the PKA-dependent troponin modulatory system, either mimicking the Ca^2+^-sensitivity shifts caused by phosphorylation or interfering with them such that Ca^2+^ sensitivity becomes independent of phosphorylation level (uncoupling).^2,5^ A well-studied example of this is the mutation in cardiac troponin C (cTnC) G159D, which causes familial DCM^6^ and has also been shown to uncouple cTnI phosphorylation from modulation of Ca^2+^ sensitivity^7−9^ (Figure 1A). Uncoupling results in a blunting of the lusitropic response to β1 receptor stimulation.^10^ Since the lusitropic response is necessary for normal heart function, suppression of lusitropy by mutations can be a significant disease mechanism in cardiomyopathy.^2,10,11^

Recent high-resolution cryo-EM images of whole thin filaments at 4.8 Å resolution combined with the fitting of structures from X-ray diffraction at 2.6 Å resolution^12−15^ can only partially describe muscle regulation. Ca^2+^ binding and switching on the thin filament are multistep dynamic processes, but current methods only show static structures. More importantly, key parts of troponin involved in contractile modulation, including the cardiac-specific N-terminal TnI extension (1–33, NcTnI), which contains the PKA phosphorylation sites, the interdomain peptide (138–147) of cTnI, and the C-terminal 18 amino acids of cTnT are not resolved experimentally, implying that they are disordered.

To understand the mechanism of regulation by troponin I phosphorylation and the effects of mutations, methods that can describe the dynamics of troponin such as NMR^16−18^ have been employed but they have their limitations since only peptide fragments of troponin can be studied. Recently, computational molecular modeling and all-atom molecular dynamics (MD) simulations have become the method of choice for understanding the phosphorylation-dependent modulation of the cardiac troponin.^19,20^

Our initial study of wild-type troponin^20^ could not ascertain with statistical significance if phosphorylation changed any of the intrasubunit interactions of cTnI. It seemed more likely that phosphorylation and mutations primarily act by altering the dynamics of the troponin molecule in ways that have not been previously analyzed.^18^

To resolve these questions, we have analyzed a substantially larger data set (5 × 1.5 μs for each system of bisphosphorylated and unphosphorylated state in the wild type and the cTnC G159D mutant) in a larger box (140 Å cube). We have identified significant phosphorylation- and mutation-dependent changes in the interatom interactions between the disordered segments of troponin and the troponin core using the Arpeggio routine.^21^ At the tertiary structure level, we observed that the principal motion within troponin was a hinge motion between the NcTnC domain and the “ITC domain”. To test the validity of the model, we correlated the changes calculated by MD with the experimentally reported effects of phosphorylation and mutation. Energetics calculations indicate that phosphorylation strengthens the TnI “switch” peptide-NcTnC interaction in the wild type. In addition, phosphorylation changes the mean TnC helix A/B angle and interdomain hinge angle distribution and restricts their range of motion. In contrast, simulation of the cTnC G159D mutation indicated that the dynamics were restricted compared with the wild type when unphosphorylated and that they changed in the opposite direction on phosphorylation, consistent with the known ability of the mutation to modulate Ca^2+^ sensitivity and block its phosphorylation-dependent change.

## Methods

The cTn model used for simulations in this project was based on that constructed and extensively studied in the group.^20^

The Ca^2+^-activated state of cTn was used for simulation, which was built upon the crystal structure of the core domain of human cTn published by Takeda et al.^12^ (PDB accession code 1J1D). The missing amino acids (the interdomain region of cTnI, residues 137–144, and the C-terminal region of TnT, residues 271–288) were added to the model using UCSF Chimera.^22^ The cardiac-specific N-terminal peptide, residues 1–33 (NcTnI), was included using the NMR structure published by Howarth et al.^23^ (PDB accession code 2JPW) in the unphosphorylated conformation. Finally, all mutations present in the crystal were changed back to the human sequence (UniProt codes: P63316, P45379, and P19429), and a redundant Ala residue present in the NcTnI NMR structure was removed. Our model contains all three regions of cTn with the full troponin C subunit and the partial structure of troponin I (1–171) and troponin T (202–288) that form the core domain of the cTn complex. The complete computational model of the core domain of cTn in the unphosphorylated state has a total of 419 residues and 6812 atoms.

The phosphorylated systems and G159D mutation were created from this model by modifying Ser22, Ser23 of the cTnI protein, and/or Gly159 of cTnC by manually editing the residues. The computational model was prepared in two different phosphorylation wild-type apo states: uP (no phosphorylation) and SEP (cTnI S22/S23 phosphorylated with net charge −2 each).

### MD Simulations

All simulations were performed using AMBER18 software suite^24^ and the CUDA-accelerated PMEMD^25^ code in the SPFP precision mode.^26^ All parameter and topology files were prepared using LEaP, which is part of AmberTools19 with AMBER ff14SB force-field library for amino acids and phosaa10 parameter library for phosphoserines (SEP).^27,28^

The simulation systems were set up by placing the protein complex in a cubic periodic boundary box with an edge length of 140 Å. The systems were first neutralized by adding Na^+^ ions as counterions and then solvated by adding water molecules (TIP3P water model^29^). To simulate physiological conditions, water molecules were replaced with ions at random to achieve a concentration of around 0.15 M NaCl. Each system underwent a five-stage minimization, heating, and equilibration process before production runs:

1. An initial minimization of the solvent for a maximum of 10,000 cycles and the protein atoms, which were restrained with a force constant of 10,000 kcal mol^–1^ Å^–2^. The minimization algorithm was switched from steepest descent to conjugate gradient after 500 steps.
2. A second minimization of all atoms in the system for a maximum of 2500 steps and switching from steepest descent to conjugate gradient after 1000 steps.
3. A first-stage heating from 0 to 100 K under an NVE ensemble with weak restraints on the protein complex (10 kcal mol^–1^ Å^–2^), except the N-terminal of TnI (1–32). The temperature was increased linearly over 50 ps.
4. A second-stage heating from 100 to 320 K over 60 ps, followed by a cooling from 320 to 300 K over 40 ps under an NPT ensemble.
5. An equilibration step of the system for 10 ns with an NPT procedure.

The minimization steps were done under constant volume with a nonbonded interaction cutoff of 12 Å. The NPT ensemble was controlled through Langevin dynamics with a collision frequency of 1 ps^–1^. A Monte Carlo barostat was used to maintain 1 bar pressure with a relaxation time of 1 ps^–1^. For the heating, equilibration, and production runs, a nonbonded interactions cutoff of 8 Å was employed and the SHAKE algorithm^30^ was used to constrain hydrogen bonds to allow for a larger time step. Hydrogen mass repartition was employed for the equilibration and production steps to allow a 4 fs time step.^31^ Five repeats were done for each system’s production simulation, and each production run lasts 1500 ns.

## MMPBSA

The molecular mechanics Poisson–Boltzmann and surface area method (MMPBSA) was used to estimate the binding affinity between segments of cTn with the rest of the protein complex. MMPBSA utilizes molecular mechanics, the implicit continuum solvent model, and the solvent-accessible surface area to estimate the enthalpy of binding.^32^ The atomic radii set for the Poisson–Boltzmann calculation was chosen to be mbondi2. MMPBSA.py,^33^ part of AmberTools, was used to carry out the calculations. No entropic contribution was calculated.

### Kernel Density Estimation (KDE) Plots

Distribution data were plotted using KDE with Seaborn (version 0.9.0).^34^ The KDE method uses kernels to represent each data point and estimates the underlying probability density function of the observed data. The default parameters and bandwidth selection methods were used for KDE plotting with Gaussian kernels.

### Atomistic Interaction Profiling

Arpeggio was used to determine the atomistic interactions between all interacting atom pairs based on atom types and positions, for all available frames.^21^ The result was aggregated to the residue level where the specific interaction type between residues was either present or not for each frame. The interaction data was summarized for all trajectories for each system to produce the interaction counts where the highest possible count is the total number of frames for each system (37500).

### Interdomain Hinge Angles

Hinge angle was defined as the angle between the regulatory domain (vector from hinge to head) and the IT arm (vector from hinge to end of the IT arm). The coordinates of the three points were defined as following:

Head: the average of C-alpha coordinates of TnC_3–85_;
Hinge: the average of C-alpha coordinates of TnC_94–157_;
End of IT arm: the average of C-alpha coordinates of TnT_241–251_, TnI_69–76_

### Helix A–B Angle

The interhelical angle was defined as the supplement of the angle between helix A (TnC 14–25) and helix B (TnC 38–47) of troponin C, similar to the convention used in previous literature.^35,36^ The end points of the helix vector were calculated by taking the average of the coordinates of 10 alpha carbons from each end of the helix. A Python reimplementation of Interhlx (K. Yap, University of Toronto) was used for the A–B angle calculations.

### Representative Structures

For each system (wild-type uP, wild-type SEP, G159D uP, and G159D SEP), the pairwise root mean square deviation (RMSD) was calculated for the five production run trajectories based on all heavy atoms. The conformation that has the lowest combined distance to all other structures within the group was picked as the representative structure. For the illustrations of interactions profiled by Arpeggio, the representative structure that most closely resembled the described interactions was chosen.

## Results

We performed extensive MD simulations of the full troponin core (419 amino acids) in the presence of Ca^2+^ in the bisphosphorylated (SEP) and unphosphorylated (uP) states for both wild-type troponin and the cTnC G159D mutant (G159D). We show results for the doubly negatively charged phosphoserine since this is the species likely to be present at physiological pH. Five 1.5 μs runs were performed for each condition, and structural and dynamic properties were analyzed.

### Dynamical Differences between Wild Type and G159D Identified by Root Mean Square Fluctuations (RMSFs)

We calculated and plotted pairwise root mean square deviation (RMSD2d) graphs for the independent simulation trajectories.^37^ We found that the variability of RMSD was essentially time independent and varied randomly between runs (Supplement Figure 1). This was also true for all the other metrics derived from the trajectories (Supplement Figures 2 and 3). We therefore combined data from all five runs in our analysis.

RMSF plots clearly identify the disordered segments of troponin, in particular the N-terminus of cTnI (NcTnI, 1–33), the cTnI interdomain peptide region (137–148), and the C-terminus of troponin T (278–288) (Supplement Figure 4). Figure 1B shows a representative structure of unphosphorylated wild-type troponin with the RMSF color-coded to illustrate the disordered regions.

For the majority of all three troponin subunits, phosphorylation results in very small perturbations of the dynamic stability of the cTnI, cTnT, and cTnC subunits, with the standard error overlapping throughout the sequence, except for a significant change in the flexibility of the NcTnI residues (1–30) with phosphorylation resulting in an approximate 33 to 50% reduction in RMSF values. The RMSFs for G159D, similarly, do not change significantly with phosphorylation and are not significantly different from the wild type.

### Arpeggio Quantitative Analysis of Wild-Type Cardiac Troponin

**Figure 2:**
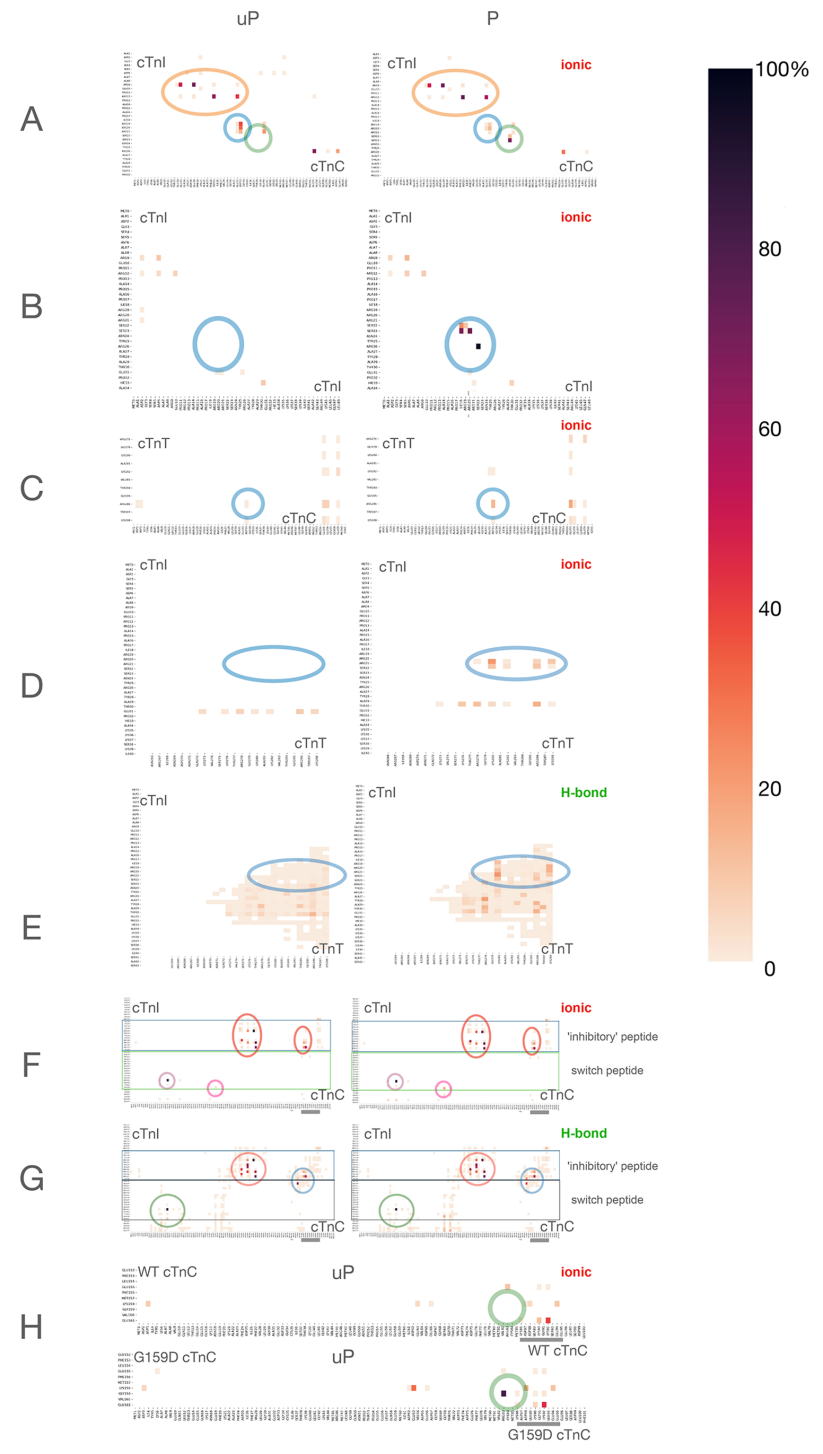
Heat maps showing the key interactions between troponin peptides related to phosphorylation. The scale represents percentage of interactions observed in 37,500 frames. Selected regions of interest are shown. The complete set of interactions are shown in Supplementary Figure 6. Numerical values for the key interactions are presented in Supporting Information Table 1 with a detailed commentary. (A) Ionic interactions between NcTnI (1–34) and NcTnC (1–84). NcTnI is anchored to NcTnC via Arg9 interacting with NcTnC Gln15 and 19 and Arg 12 interacting with NcTn C Asp25 and Glu32 (orange circle). A strong interaction between NcTnC Lys33 and NcTnI Arg19, 20, and 21 is lost on phosphorylation (blue circle), while interaction with NcTnC Lys39 is gained (green circle). (B) Ionic intramolecular interaction within NcTnI (1–34 vs 1–48). Phosphorylation promotes interaction of phosphoserine 22 with Arg19, 21, and 26 (blue circle). (C–E) Key interactions of CcTnT (C) ionic interactions between CcTnT (280–288) and NcTnC (1–46). There is a medium interaction formed between NcTnC Asp33 and Arg286 upon phosphorylation (blue circle). (D) Ionic interactions between CcTnT (281–288) and NcTnI (1–40). Upon phosphorylation, CcTnT, especially Lys288, interacts with TnI Ser 22 and 23 (blue circle). (E) H-bond interactions between CcTnT (266–288) and NcTnC (1–40). Phosphorylation increases H bonding between NcTnI Arg 19, 20, and 21 and CcTnT Lys280, 287, and 288 (blue circle). (F, G) Ionic and H-bonding interactions of the switch peptide and cTnI interdomain peptides with cTnC. The switch peptide is outlined in black; the “cTnI interdomain” peptide is outlined in blue. The TnC interdomain peptide (86–95) is represented by a gray bar. (F) Ionic interaction between cTnI (126–170) and cTnC (1–109). The switch peptide is anchored by a stable interaction between NcTnC Glu19 and cTnI 161 (green circle). The interaction of NcTnC Lys43 with cTnI 164 and 169 and the interaction of cNcTnc Arg46 with cTnI Ala170 are decreased on phosphorylation (pink circle). Altered interactions of the cTnI interdomain peptide with NcTnC helices C and D are shown in the blue circle: Asp62 and Glu63 interaction with cTnI Arg140 and 145 increases, while interaction of Glu56 with cTnI Arg144 and 145 decreases. Altered interactions of the cTnC linker peptide with the cTnI interdomain peptide upon phosphorylation are indicated by the orange circle. (G) H-bond interactions between cTnI (126–170) and cTnC (1–109). The switch peptide is anchored by a stable interaction between NcTnC Glu19 and cTnI 161 (green circle). Increase of the interactions of cTnC 84 and Ser89 with cTnI Ala150 and Gly159 is highlighted by the blue circle. (H) Comparison of ionic interaction between cTnC (152–161) and cTnC (1–100) in unphosphorylated wild type and G159D. The most significant change from the wild type is the formation of a strong, ∼80%, ionic interaction between Arg83, at the bottom of the D helix and the Asp159.

**Figure 3:**
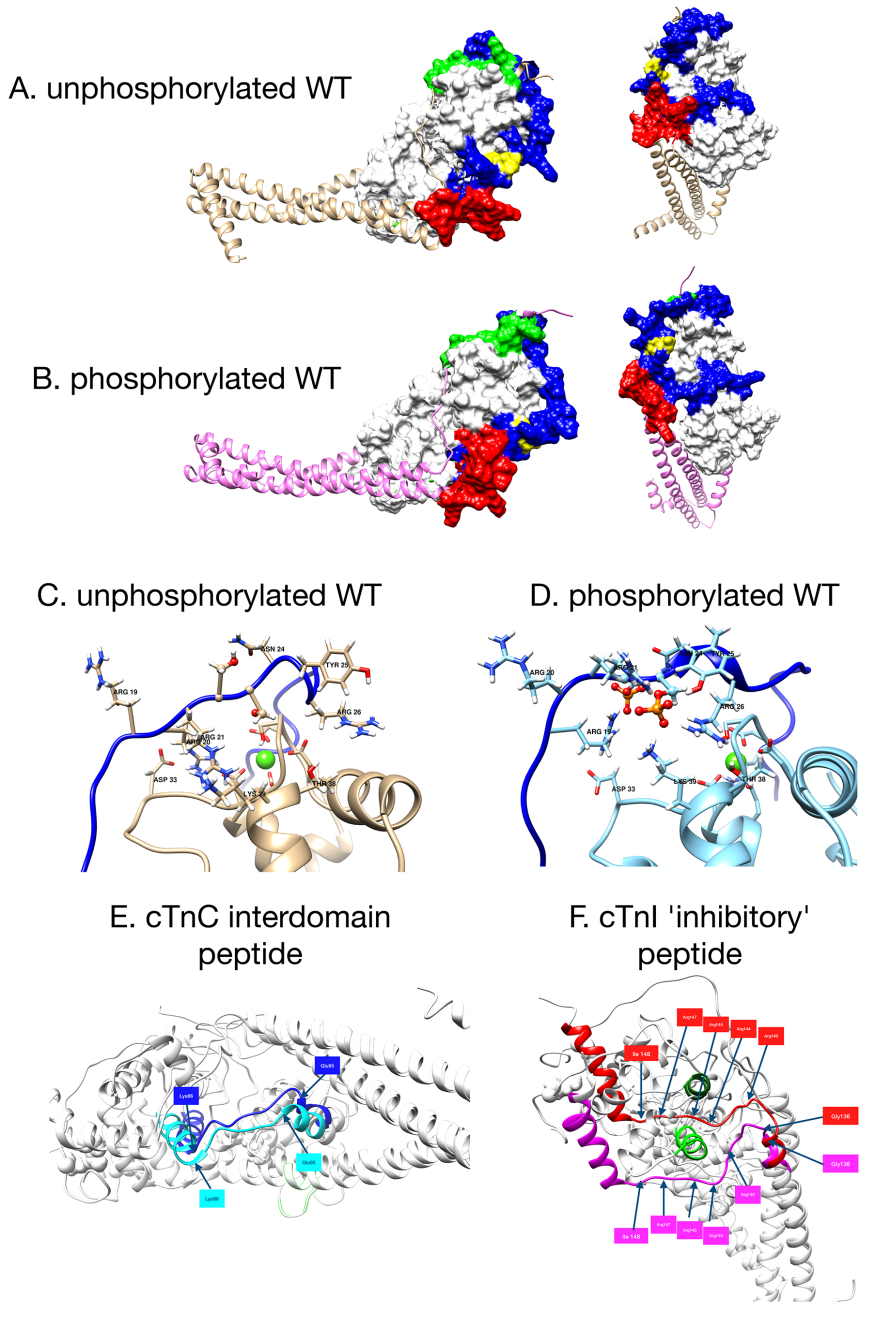
Representative structures of the cardiac troponin core for wild type and G159D in the unphosphorylated and phosphorylated states. (A, B) Representative structure of wild-type cardiac troponin with surface rendering of NcTnC (white), NcTnI (blue), switch peptide (green), and CcTnT (red). Views are from the side and the “top” of troponin. Rotating models are presented as mp4 movies, and the PDB files used to generate the structures are presented in the supplement. (A) Unphosphorylated, (B) phosphorylated. (C, D) Partial model showing the key phosphorylation-dependent interactions between NcTnI and NcTnC only. TnI Arg19–Arg26 and TnC Asp33, Thr38, and Lys39 are shown in stick format. Rotating models are presented in the supplement. (C) Unphosphorylated, (D) phosphorylated. (E) The interdomain peptide of cTnC in unphosphorylated (cyan) and phosphorylated (blue) states. Phosphorylated and unphosphorylated representative structures are aligned to the IT coiled coil. The interdomain peptide (86–95), helix D, and part of helix E are highlighted to illustrate the lengthening and twisting of the peptide upon phosphorylation. Distribution of interdomain peptide lengths is shown in Supplement Figure 5 and quantified in Table 1. (F) The cTnI interdomain peptide (136–148) and switch peptide (149–164) are highlighted in unphosphorylated (magenta) and phosphorylated (red) states. TnC helix C is highlighted in light green (uP) and dark green (P). Phosphorylated and unphosphorylated representative structures are aligned to the IT coiled coil. The model is oriented to show the change in the NcTnc-ITC domain angle.

We have used the Arpeggio package^21^ to quantify phosphorylation and mutation-related differences. Arpeggio identifies and classifies interactions between atoms based on proximity and angles. We then summarized the counts for H-bond, ionic, aromatic, and van der Waals interactions between all residue pairs. Full details of this analysis are given in Methods. Applying this analysis upon all 37,500 frames of a data set facilitates quantitative measurement of significant changes in interactions between the different systems due to phosphorylation and mutations. Supplementary Table 1 shows the numerical stability of the interactions that change significantly with phosphorylation or mutation with a detailed analysis, and complete results are in Supplementary Data Set 1. The interaction counts are illustrated in heat maps (Figure 2, Supplement Figure 6), and representative structures are shown in Figure 3.

Residues 1–35 of NcTnI, including phosphorylatable serines 22 and 23, are positioned over the NcTnC domain (Figures 3A–D). Addressing first the phosphorylation-independent interactions, we find that NcTnI is anchored on TnC, near the N-terminal end by interactions between NcTnI Arg9 and glutamines 15 and 19 of NcTnC, which are the start of cTnC helix A (orange circle, Figure 2A).

Phosphorylation significantly modulates cTnI–cTnC, cTnI–cTnI, and cTnI–cTnT interactions. In the unphosphorylated troponin, the major interaction between NcTnC and NcTnI is cTnC Asp33, in the EF hand loop I, with cTnI Arg19, 20, and 21 (blue circle, Figure 2A). This is significant as Asp33 is one of the few cardiac-specific variants in cTnC (Gly33 in skeletal muscle cTnC). Upon phosphorylation, there is a cumulative loss of these interactions (−31, −20, and −14%, respectively). This is accompanied by altered NcTnC–NcTnI interactions with increased cTnC Asp25 to cTnI Arg12 interaction and the cTnC Glu32 to cTnI Arg12 interaction (+15%), both cTnC residues being at the end of the A helix.

Interactions of Ser22 and Ser23 with cTnC Lys39 are formed upon phosphorylation. The ionic interactions with Ser22 and 23 increase from 0 to ∼80% of the simulations (green circle). The resulting shift of NcTnI–NcTnC interactions changes the location of NcTnI on NcTnC and results in Lys39 in the B helix being pulled toward the phosphorylated serines of NcTnI, confirmed by the formation of a hydrogen bond interaction between cTnC Thr38 and cTnI (+32%). As a consequence, there is also a very noticeable change in the orientation of the end of helix B relative to the A helix. There is a loss of interaction of NcTnC Glu40 with NcTnI Arg20/21 (−6%/–20%). Uniquely, there is a strong phosphorylation effect on the aromatic interaction of the phenylalanine residues in the A and D helices. While the interaction between the end of the A helix Phe24 with the start of the D-helix Phe74 and 77 is relatively constant, the very end of the A helix Phe27 increases its interaction upon phosphorylation with the D-helix Phe77 (+13%).

At the same time, new intra-NcTnI interactions are formed between the phosphate groups of Ser22 and Ser23 and arginines in NcTnI (Figures 2B and 3D). There is a very clear preference for Ser23 to form an ionic interaction with cTnI Arg19, 21, and 26 (+68, +72, and +95%, respectively), while only Arg19 makes significant contact with phosphorylated Ser22 and Arg20 only interacts weakly in the phosphorylated state.

The Arpeggio analysis reveals interactions between the C-terminus of cTnT and both cTnC and cTnI not previously noted (Figure 2C–E). There is a medium interaction formed between the NcTnC EF1 hand, Asp33, and the CcTnT Arg286 upon phosphorylation (0 > 25%), which correlates with the repositioning of the cTnC Lys39 and the phosphorylated serines described previously.

There are H-bond interactions between the CcTnT Lys280 and the phosphorylated Ser22 and Ser23 of NcTnI that are completely absent in the unphosphorylated state (Figure 2D). There is also an increased cumulative ionic interaction of 10–20% between cTnI Arg19, 20, and 21 and CcTnT Lys280 and 288 upon phosphorylation, which, like the interaction with Ser22 and 23, is absent in the unphosphorylated state (Figure 2E).

The troponin I switch peptide, 149–164, is docked onto the hydrophobic patch of NcTnC formed by helices A and B. It is anchored to NcTnC by a stable interaction between NcTnC Glu19 and cTnI Arg161, but the repositioning of cTnC helix B upon phosphorylation alters the hydrophobic patch structure and results in its repositioning. There is a noticeable loss of interactions between cTnC helix B and the C-terminal part of the switch peptide. The interaction of NcTnC Lys43 with the switch peptide residues Glu164 (−16%) and Asp167 decreases, and the interaction of cTnC Arg46 with Glu164 and Asp167 also decreases. At the N-terminal end of the switch peptide, there is around 12% increased hydrogen bonding between cTnC Cys84 and cTnI Ala150 and cTnC Ser89 and Gly91 with cTnI Gly159 (Figure 2F,G).

The N-terminal domain of troponin C is linked to the ITC domain by two unstructured peptides that cross the interface: cTnC 86–95 and cTnI 136–148. The configuration of the two peptides defines the relative orientations of the two domains. Rearrangement of cTnC helix B on phosphorylation alters helices C and D. On phosphorylation, contact between the end of D-helix Arg83 and residues Asp88 and Glu155 is weakened, with the linker moving away from the D helix. At the same time, the linker region, Asp88 and Gly91, moves collectively toward CcTnC, accompanied by changes in the E helix. At the start of the E helix, Ser93 is moving away from the rest of the helix. This is reflected in the reduction of its interactions with Glu95, Glu96, and Leu97 (−20, −22, and −13% respectively). The overall effect is that the cTnC linker peptide lengthens upon phosphorylation increasing the distance between the NcTnC and CcTnC domains (Figure 3E). The increased distance is quantified in Table 1 and Supplementary Figure 5.

**Table 1:** Effects of Phosphorylation and the G159D Mutation on Structural Metrics of the Cardiac Troponin Core Determined by Molecular Dynamics Simulations^a^

		wild type			G159D		
		uP	P	Δ	uP	P	Δ
**parameter**	**metric**						
**interdomain hinge angle**	mean (std)	121.61° (7.24)	123.26° (5.95)	+1.65	121.51° (4.82)	116.2° (6.04)	–5.31
	KDE	121.9°	122.5°	+0.6	121.7°	117.1°	–4.6
	FWHM	22.2°	16.6°	–25%	11.6°	12.4°	+7%
							
A/B helix angle	mean (std)	101.8° (9.15)	96.16° (5.73)	–5.64	97.06° (5.48)	98.97° (6.73)	+1.9
	KDE	96.3°	95°	–1.3	95.7°	95.4°	–0.3
	>110°	18%	1%		1%	7%	
	FWHM	17.1°	12.3°	–28%	12.6°	13.8°	+10%
							
**distance between NTnC and CTnC**	mean (std)	30.18 Å (1.34)	29.90 Å (0.9)	–0.3	29.22 Å (0.98)	29.59 Å (1.18)	+0.37
	KDE	31.1 Å	29.6 Å	–0.5	29.0 Å	29.2 Å	+0.2
	FWHM	2.98 Å	2.39 Å	–0.59	1.58 Å	1.54 Å	–0.04
							
**mean MMPBSA (std), Δ*G*, kcal/mol**	**switch peptide**	–108.4 (24.1)	–111.9 (13.9)	–3.5	–113.5 (13.8)	–117.0 (15.0)	–3.5
	**cTnI interdomain peptide**	–153.7 (23.4)	–157.6 (20.1)	–3.9	–168.5 (23.8)	–164.9 (20.5)	+3.6
	**TnI**_**34–71**_	–215.7 (21.2)	–226.2 (20.1)	–10.5	–212.4 (19.9)	–217.3 (19.9)	–4.9

a The distance between NTnC and CTnC is measured as the distance between the center of NTnC (average coordinates of C-alpha of TnC 3–85) and the center of CTnC (average coordinates of C-alpha of TnC 94–157). KDE: kernel density estimation. FWHM: full width at half-maximum peak. MMPBSA: molecular mechanics Poisson–Boltzmann and surface area. std: standard deviation.

Repositioning of the cTnI switch peptide relative to cTnC helices A and B on phosphorylation alters the adjacent cTnI interdomain peptide, 136–148. The cTnC C helix moves with the B helix, thus altering its interactions with the middle of the cTnI interdomain peptide. The arginines in this peptide (140, 144, 145, 147, 149) form ionic interactions with cTnC, notably at the start of helix C and the end of helix D that shift on phosphorylation. Overall, upon phosphorylation, the cTnI interdomain peptide repositions toward the start of C helix (Figure 3F).

### Arpeggio Quantitative Analysis of G159D Mutant Cardiac Troponin

Examining first the effects of the G159D mutation on cTnC, which are phosphorylation independent, the most significant change from the wild type is the formation of a strong ionic and hydrogen bond interaction between Arg83, at the bottom of the D helix, and Asp159, at the end of the H helix (∼80% of the simulations). Arg83 also forms a strong ionic interaction with the N helix of cTnC, which is phosphorylation dependent in the wild type (Figure 2H, Supplement Figure 6G).

Structural differences accompanying phosphorylation of G159D, in both magnitude and sign, can be observed with respect to the interaction of the N helix of cTnC and the beginning of the H1 helix of NcTnI (Supplement Table 1, Supplement Figure 6 H,I). The key phosphorylation-dependent interactions between cTnI Arg19, Arg21, Ser22, Ser23, and Arg26 with cTnC Lys33 and Lys39 change similar to the wild type. However, reversal of the observed changes in interactions upon phosphorylation for G159D relative to the wild type is frequent in the region of the interdomain interface. Thus, the ionic interaction of cTnC Asp25 and Glu32 with cTnI Arg12 in the G159D phosphorylated state decreases from the unphosphorylated state (−14%) while in the wild type they increase (+7%). This is particularly evident in the interaction of the linker region of cTnC with itself; for residues Lys90 with Glu94, the ionic interactions increase upon phosphorylation (+3%), while for the wild type, they decrease (−6%). The collective effect in the linker region of G159D is that phosphorylation induces a shortening of the linker, which is the direct opposite of the observation in the wild type (see Table 1, Supplement Figure 5). This reversal of behavior upon phosphorylation of G159D relative to the wild type is also observed in the interactions of the EF3 hand of TnC with CcTnT (Supplement Table 1).

### Global Changes in Troponin Dynamics due to Phosphorylation and Mutation

**Figure 4:**
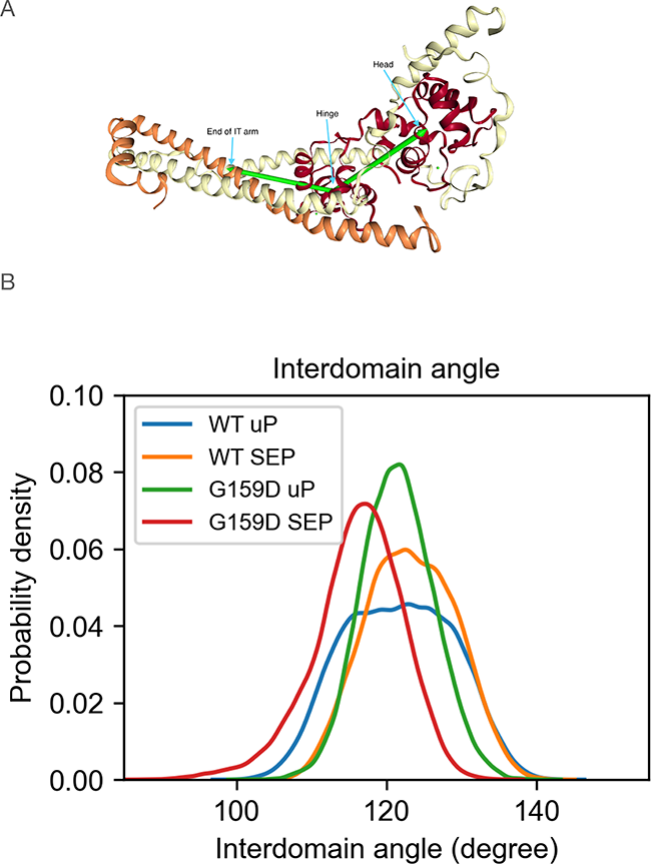
(A) Definition of the interdomain angle. Head: the average of C-alpha coordinates of TnC_3–85_; fulcrum: the average of C-alpha coordinates of TnC_94–157_; end of IT arm: the average of C-alpha coordinates of TnT_241–251_ and TnI_69–76_. (B) Probability distribution of the angle between the NcTnC and ITC domains.

The MD simulations demonstrate that the principal motion is a hinge motion between the N-terminal domain of troponin C and the ITC arm. We investigated whether phosphorylation and mutation affected hinge dynamics (Figure 4 and Table 1). In the wild type, the mean hinge angle increases on phosphorylation from 121.6 to 123.3°, whereas in the presence of the G159D mutation, the mean angle decreases from 121.5 to 116.2°. The same pattern is observed using the angle of the highest density from the KDE of the data (Table 1). Figure 4B shows the distribution of angles and confirms the trend toward populations with a higher angle on phosphorylation of the wild type and the opposite trend for G159D. The distribution of angles, as quantified by the full width at half-maximum (FWHM) values, is also phosphorylation dependent (Table 1). The unphosphorylated wild type has a very broad distribution of accessible angles, which upon phosphorylation is *reduced* by ∼25% while G159D has a much reduced range in both states. In unphosphorylated G159D, the angle is ∼25% less than the phosphorylated wild type, while upon phosphorylation there is an *increase* of about 7%.

**Figure 5:**
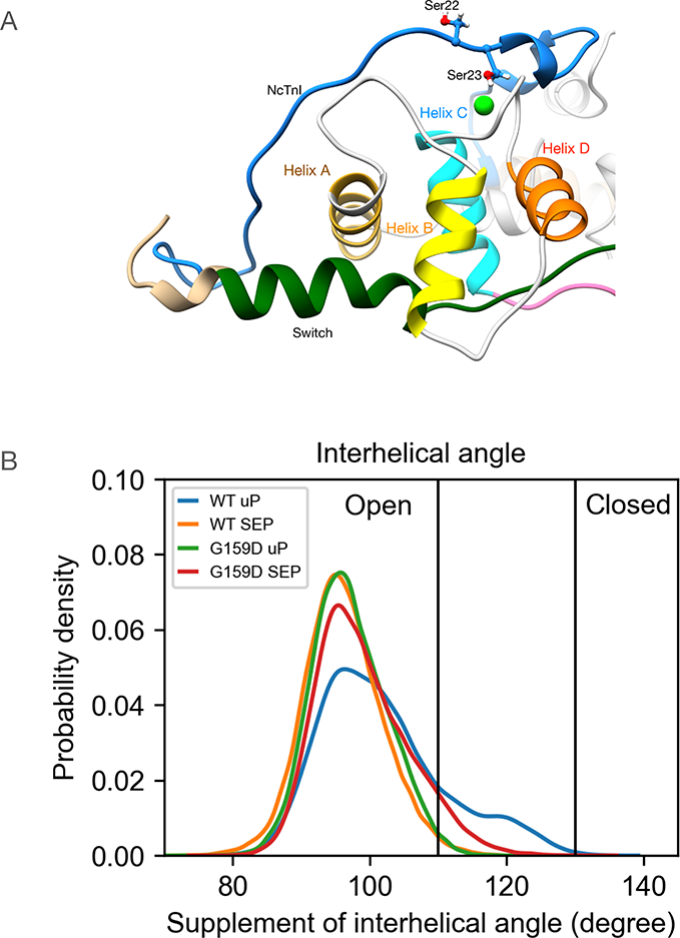
(A) Model of the backbone structure of NcTnC showing the relative disposition of helices A and B in the unphosphorylated state. (B) The distribution of the interhelical angle formed by helices A and B in the unphosphorylated and phosphorylated (SEP) states for the wild-type and cTnC G159D-mutant troponin. The definition of open and closed angles is adopted from Lindert et al.^36^

The angle between helices A and B of NcTnC (A/B angle) has previously been identified as a suitable metric for evaluating the effect of Ca^2+^ activation upon troponin.^35^ In Figure 5A, we illustrate the disposition of the helices relative to each other, using the definition of the angle between them given by Kekenes-Huskey et al.^36^ For all our systems, the helix A/B angle is predominantly in the “open” conformation characteristic of Ca^2+^-liganded troponin with the switch peptide docked.

Figure 5B shows the distribution of angles, which are quantified in Table 1. For the wild type, the effect of phosphorylation results in a decrease of the mean A/B angle, from 101.8 to 96.1°. For the G159D mutant, we observe the complete inverse behavior: Upon phosphorylation, the mean A/B angle increases from 97.1 to 99.0°. Additionally, comparing the wild type and G159D, unphosphorylated A/B angles, we see that the wild-type angle is higher at 101.8° compared with G159D at 97.1°. This is further reinforced when considering the percentage of the area under the curve for the A/B angle above 110°, which is the angle assigned to be the “open” conformation threshold by Kekenes-Huskey et al.^35,36^ For the unphosphorylated wild type, this is 18%, and for G159D, it is 1%; the corresponding values for the phosphorylated states are 1% (wild type) and 7% (G159D). The A/B angle of highest density using KDE, however, indicates that while there is a small difference between wild-type unphosphorylated and phosphorylated in the same direction as the mean angle value, there is only the smallest of differences between the phosphorylated wild type and G159D in both states. The FWHM parameter (Table 1) demonstrates significant phosphorylation- and mutation-dependent differences in behavior. For the wild type, FWHM is reduced ∼28% upon phosphorylation, very similar to the reduction observed in the hinge angle. For G159D, phosphorylation results in a 10% increase in the FWHM to 13.8°. Significantly, the unphosphorylated G159D and wild-type phosphorylated are comparable at 12.6 and 12.3°, respectively.

The interaction energy between regions of cTnC and cTnI has been evaluated using the MMPBSA methodology (Table 1). Three regions of cTnI were chosen due to their structural and functional significance: the switch peptide (cTnI_147–163_), which is in close contact with the A/B angle (hydrophobic patch) region of NcTnC; cTnI_34–71_, which interacts with the C-terminal domain of cTnC and is close to the G159D mutation site; and finally, the cTnI interdomain peptide (TnI_131–147_), which crosses from the NcTnC domain to the ITC domain and is hypothesized to be critical in the contraction/relaxation process. The distribution of MMPBSA values is shown in Supplementary Figure 7.

We found that the switch peptide binds to the hydrophobic patch more strongly upon phosphorylation, with similar ΔΔ*G* values for the wild type and G159D mutant. This result correctly reproduced the trend found by the wild-type NMR titration; comparing the difference in Δ*G* upon phosphorylation for the wild type, there is, perhaps fortuitously, a similarity in the magnitude between experiment and simulation, ΔΔ*G* = −1.2 and −3.5 kcal/mol, respectively.^38^

The cTnI interdomain peptide binding affinity of the wild type was observed to increase on phosphorylation (ΔΔ*G* = −3.9 kcal/mol); conversely, there was decreased affinity in the TnC G159D mutant (ΔΔ*G* = +3.6 kcal/mol).

Troponin C is anchored in the complex through an interaction of the C-terminal domain with TnI_34–71_, and the G159D mutation site is located at the C terminal of cTnC. We found that the interaction between cTnI_34–71_ and the rest of the troponin complex is slightly weaker in G159D compared with the wild type (G159D uP −212.4 kcal/mol vs wild-type uP −215.7 kcal/mol), in agreement with previous measurements.^39^ In the phosphorylated state, wild-type and G159D mutant cTnC both showed a higher affinity for the cTnI_34–71_ region than in the unphosphorylated state, being more marked in the wild type.

MD simulations produce a large number of correlated samples for every metric. The distributions are often non-Gaussian; therefore, traditional statistical tests are not appropriate. Effect size is a more descriptive metric in that it qualitatively describes how large the difference is between the two samples. Traditional statistical tests tend to deal with experimental data with a low number of observations. This means that most of the comparisons stemming from this work are likely to produce significant test statistics when the null hypothesis is that the mean of the two distributions is different although the actual difference could be negligible. Effect size is a more descriptive metric in that it qualitatively describes how big the difference is between the two samples. Here, we used Cohen’s *d* to assess the effect size between the means of the data sets when making comparisons (Supplementary Table 2). Low values often correlate with bimodal distributions

## Discussion

The phosphorylation of serines 22 and 23 of troponin I in cardiac troponin modulates the myofilbrillar Ca^2+^ sensitivity and increases the rate of Ca^2+^ release from troponin C. It is a major determinant of the lusitropic response to adrenergic stimulation^10,40^ The molecular mechanism of this process has remained unknown long after the mechanism of the troponin Ca^2+^ switch itself was defined.^12,13,41^ Investigation of this subtle process at the atomic level poses a challenge for structural biology since the change in Ca^2+^sensitivity is only about twofold,^42^ and key parts of the troponin modulation and regulation system are disordered and cannot be fully resolved by most methodologies. In this study, we have used MD simulations of the cardiac troponin core to understand the molecular mechanism by which phosphorylation of serines 22 and 23 and a DCM-linked mutation, cTnC G159D, modulate Ca^2+^ regulation in cardiac muscle.

In previous studies of troponin using MD methods, conclusions were difficult to sustain due to questions about the box size used and the length of simulations.^19,20,43^ Our extended simulations using a 140 Å cubic water box has avoided these problems and now allows us to demonstrate that Ca^2+^-saturated troponin exhibits a considerable range of motion and that NcTnI, the cTnI interdomain peptide, and the CcTnT can fluctuate over a range of conformations. The N-terminal peptide of troponin I is located on the surface of the N-terminal domain of troponin C. Phosphorylation and mutations alter the dynamics of troponin, and by using the Arpeggio technique, we have been able to define significant local changes in the populations of these conformations that could explain the action of phosphorylation and mutations.

### Local Changes Responsible for Large-Scale Conformational Changes

The direct effect of phosphorylation at serines 22 and 23 is a local rearrangement in which basic amino acids, including neighboring arginines in NcTnI (Arg19, 21, and 26) and cTnC lysine 39 tend to form a cluster of ionic bonds with the phosphate groups of phosphoserines 22 and 23 at the expense of interactions between cTnI and cTnC, particularly the cTnC Asp33 interaction with Arg19, 20, and 21 of NcTnI. The pulling of cTnC Lys39 toward cTnI serines 22 and 23 impinges upon helix B and its orientation relative to helix A and the hydrophobic patch that the switch peptide binds to, manifested as the change in interhelical angle and increased affinity of the switch peptide for cTnC. Longer-range allosteric consequences of the helix B rearrangement include a repositioning of the switch peptide and changes in the peptides crossing the interdomain interface that can account for the observed changes in hinge angle.

There is considerable rearrangement and lengthening of the interdomain “linker” region of cTnC, residues 87–97, upon phosphorylation. This has an effect on the disposition of the NcTnC domain relative to that of the CcTnC domain, with the two domains becoming closer. There are also changes in the interaction of the CcTnT and the EF3 and 4 hands of CcTnC resulting in the close coupling of CcTnT and CcTnC.

Finally, there is also a strong local change in the positioning of the cTnI interdomain peptide (cTnI residues 139–148) relative to the C and E helices of cTnC. Upon phosphorylation, the cTnI interdomain peptide moves to position itself closer to the start of the cTnC C helix, losing its interaction with the CcTnC E helix.

### Global Changes Calculated by MD Correlate with Changes in Experimentally Measured Parameters

The local changes in troponin described above lead to macroscopic changes that can be compared with experiment. We have based our analysis on the consensus model of Ca^2+^ switching, as summarized by Stevens et al.^44^ Ca^2+^ activation of troponin is a two-step process: first, Ca^2+^ binds to site II of troponin C, and then the hydrophobic patch of cTnC opens (energetically unfavored transition state) and the switch peptide binds to the open patch forming the active state.^3,9^ Since our studies are all in the presence of Ca^2+^ bound, we only see the active (“open”) state.

The principal motion of troponin is a hinge-like motion between the two quasi-rigid domains of troponin, NcTnC and the ITC domain.^20,13^ The hinge angle is altered by Ca^2+^ activation^45^ and is also proposed to be modulated by phosphorylation^17,18,46^ The simulations indicate that upon phosphorylation, the distribution of hinge angles in wild-type troponin shows an increase in the mean angle with a significant rigidification and decrease of the accessible range. This is consistent with the studies of Hwang et al.,^17^ who proposed that phosphorylation modified the positioning of NcTnC relative to the ITC domain, based on NMR measurements. It is notable that the C terminus of troponin T (CcTnT), previously not included in MD simulations,^19^ is located within the interdomain interface where its interactions with NcTnI and cTnC are phosphorylation dependent, thus possibly accounting for the known role of this peptide in cTn regulation^47,48^

Using the A/B helix angle as our metric for the opening and closing of the hydrophobic patch, we found that, indeed, phosphorylation has a significant effect on the distribution of interhelix angles with an increase from 82 to 99% distribution below 110°, which was proposed as the threshold of the “open” state. Accompanying the hinge angle change, there is a significant rigidification and decrease in the accessible range upon phosphorylation.

Finally, we note that interaction energy calculations from MD simulations indicate that phosphorylation strengthens the interaction between the cTnC hydrophobic patch and the cTnI switch peptide in quantitative agreement with Baryshnikova et al. using NMR titrations.^38^

In summary, the global effect of phosphorylation on the dynamics of the wild type is comparable with experimental measurements of changes in the interdomain hinge angle, A/B helix angle, and ΔΔ*G* switch peptide. MD shows us that phosphorylation uniformly reduces the accessible range of whichever metric we observe, suggesting that phosphorylation results in rigidification of the overall structure of the troponin complex.

### Effect of DCM-Linked TnC G159D Mutation on Troponin Dynamics

To test further the correlation between experimental data and the changes in troponin dynamics due to phosphorylation, we studied the cTnC G159D mutation, which is associated with familial DCM. The key effect of the mutation is that it abolishes the shift in Ca^2+^ sensitivity due to PKA phosphorylation of serines 22 and 23 that is crucial for the lusitropic effect,^7,8^ and this may be sufficient to cause the DCM phenotype.^11^ We found that the G159D mutation has significant effects upon the dynamics of troponin.

The primary difference due to the G159D mutation in cTnC is the formation of strong and phosphorylation-independent bonds across the bottom of the interdomain interface, particularly between cTnC Arg83 and Asp159; consequently, the range of hinge angles is reduced. This affects the location of the apparent fulcrum of the “hinge” so that the phosphorylation-dependent changes in the peptides crossing the interdomain interface can result in opposite changes consistent with the functional abnormalities caused by the mutation.

Unphosphorylated G159D troponin has a lower mean A/B helix angle than the unphosphorylated wild-type troponin and a higher probability of being in the range of angles defined as the open state. The mean hinge angle is similar to that of the phosphorylated wild-type troponin, but the distribution is narrower. In general, unphosphorylated G159D resembles the phosphorylated wild type more than the unphosphorylated wild type.

Interestingly, the effect of phosphorylation on these metrics is in the opposite direction of the wild type. For instance, for the A/B metric, phosphorylation of G159D increased the mean angle by 1.9° and decreased the proportion below 110° (open state threshold) from 99 to 93%. Phosphorylation of G159D also shifts the interdomain angle distribution toward a lower mean angle and increased RMSF across the whole system, particularly troponin C, indicating a global as well as the specific effect of the point mutation. The findings that the wild-type response to phosphorylation, observed in our MD simulations, is disrupted by the G159D mutation correlates with the observation that the G159D mutation abolishes the shift in Ca^2+^ sensitivity due to PKA phosphorylation of Ser22 and 23.^7,8^ It is noticeable that the interactions between NcTnI and NcTnC involving cTnC Lys 33 or Lys39 and TnI Ser 22 and 23 and the arginines 19, 20, 21, and 26 are the same as the wild type and change in the same way on phosphorylation, independent of the mutation-related changes in the interdomain region. This indicates conformational uncoupling that mirrors the functional uncoupling caused by the mutation.

## Conclusions

The comparative results support our proposal that the MD simulations faithfully report relevant changes in dynamics induced by phosphorylation and mutation of the troponin core. We observed limited phosphorylation-dependent changes in the equilibria between a range of conformations of the troponin core similar to those known to be on the Ca^2+^ activation pathway of troponin, consistent with an enhanced probability of the open state on phosphorylation. Phosphorylation results in rigidification of the overall structure of the troponin complex. We suggest that this stabilizes cTnI switch peptide docking to the hydrophobic patch of troponin C in the Ca^2+^-bound (open) state, and this has an effect on Ca^2+^ sensitivity and Ca^2+^ release. The observation that an uncoupling mutation induces an opposite change in these metrics on phosphorylation supports this proposition. This could be further tested with other uncoupling mutations or small molecules that can reverse uncoupling;^49^ it is noteworthy that most of the mutations in the troponin core that are reported to uncouple are at the interdomain interface (Supplement Figure 8). Rigidification of the overall structure of troponin by mutations, mimicking phosphorylation, may explain how many different mutations in various components of the thin filament can uncouple^2,50^

*In vivo* troponin is incorporated into the thin filament and interacts with tropomyosin and actin. The troponin core has been docked into recent high-resolution cryo-em images of the thin filament at both relaxed and activating Ca^2+^ levels, and the positions of the cTnI N terminus and cTnT C terminus, not included in the troponin core, have been resolved. Nevertheless, cryo-em still does not provide any information about the disordered segments of troponin described here. An attempt has been made to model the NcTnC peptide into the em envelope and suggests there may be additional interactions with tropomyosin.^51^ With the new thin-filament structural models as a starting point and with ever-increasing capability of processors for MD calculations, an all-atom simulation of the thin filament, including the disordered regions, has become feasible.

## Supporting Information Available

2D RMSD plots of simulation trajectories; timelines for the hinge angle parameter for all the 1500 ns runs; timelines for the helix A/B angle parameter for all the 1500 ns runs; root mean squared fluctuations (RMSFs) of the individual troponin subunits; distribution of length of the cTnC "linking peptide"; heat maps of peptide–peptide interactions, calculated from the data in Supplementary Table 1; distribution of MMPBSA values; the location of mutations in the core domain of troponin that have been shown to uncouple Ca^2+^ sensitivity from TnI phosphorylation; Arpeggio results showing main changes due to phosphorylation and mutation; and Cohen’s *d* calculated for structural and energetic parameters (PDF)
Representative structure of wild-type cardiac troponin with surface rendering of NcTnC, NcTnI, switch peptide, and CcTnT—unphosphorylated (MOV)
Representative structure of wild-type cardiac troponin with surface rendering of NcTnC, NcTnI, switch peptide, and CcTnT—phosphorylated (MOV)
Partial model showing the key phosphorylation-dependent interactions between NcTnI and NcTnC only—phosphorylated (MOV)
Partial model showing the key phosphorylation-dependent interactions between NcTnI and NcTnC only—phosphorylated (MOV)
Complete Arpeggio data and PDB files of representative structures referred to in the text (ZIP)
G159D troponin, phosphorylated (MOV)
G159D troponin, unphosphorylated (MOV)

## Supplementary Material

jp3c02337_si_001.pdf

jp3c02337_si_002.mov

jp3c02337_si_003.mov

jp3c02337_si_004.mov

jp3c02337_si_005.mov

jp3c02337_si_006.zip

jp3c02337_si_007.mov

jp3c02337_si_008.mov
