# Supplementary material for: Modulation of Structure and Dynamics of Cardiac Troponin by Phosphorylation and Mutations Revealed by Molecular Dynamics Simulations
Source: J Phys Chem B. 2023 Oct 4;127(41):8736–48. doi: 10.1021/acs.jpcb.3c02337 (PMC10591477; doi:10.1021/acs.jpcb.3c02337)
Supplement: Supplementary file 1 — jp3c02337_si_001.pdf [file jp3c02337_si_001.pdf]

Supplementary material for

TITLE: **Modulation of structure and dynamics of cardiac troponin by phosphorylation and mutations revealed by molecular dynamics simulations**

AUTHORS: Zeyu Yang[1], Stephen B. Marston[2] and Ian R. Gould[1,]*

1. 1) Department of Chemistry, Imperial College London, Molecular Sciences Research Hub, Shepherd's Bush, London, W12 0BZ, UK and the Institute of Chemical Biology, Imperial College London, Molecular Sciences Research Hub, Shepherd's Bush, London, W12 0BZ, UK
2. 2) National Heart & Lung Institute, Imperial College London, W12 0NN, UK

Zeyu Yang https://orcid.org/0000-0002-2957-8063 Steve B Marston https://orcid.org/0000-0001-6054-6070 Ian R Gould https://orcid.org/0000-0003-3559-0234

**corresponding author e-mail i.gould@imperial.ac.uk*

Supplementary data FIGURES Supplementary figure 1

2D RMSD plots of simulation trajectories comparing frames within each trajectory. No consistent time-dependent large scale conformational changes were observed from the beginning of simulations.
(see Grossfield, A. *et al.* Best Practices for Quantification of Uncertainty and Sampling Quality in Molecular Simulations [Article v1.0]. *Living J Comput Mol Sci* **1**, (2019))

Supplementary Figure 2

Timelines for the hinge angle parameter for all the 1500ns runs

Supplementary Figure 3

Timelines for the helix A/B angle parameter for all the 1500ns runs

Supplementary figure 4

Root Mean Squared Fluctuations (RMSF) of the individual troponin subunits. Wild-type (left) and TnC G159D mutation (right) in unphosphorylated (blue) and phosphorylated (orange) states. The schematics below indicate the structural elements of TnI, TnT and TnC based on Takeda's notation. The disordered regions are ringed. Numerically, the RMSF's are lower with the ff14SB force field compared with previous studies using ff99SB.

Supplementary figure 5

Distribution of length of cTnC 'linking peptide'

Supplementary figure 6

Heat maps of peptide-peptide interactions, calculated from the data in supplementary table 1

A TnI-TnC ionic interactions, uP and P compared
B TnI-TnI ionic interactions, uP and P compared
C TnI-TnC H bond interactions, uP and P compared
D TnT-TnC ionic interactions, uP and P compared
E TnT-TnI ionic interactions, uP and P compared
F TnT-TnI H bond interactions, uP and P compared
G TnC-TnC ionic interactions, uP WT and uP G159D compared
H TnI-TnC G159D ionic interactions, uP and P compared
I TnC-TnC G159D ionic interactions, uP and P compared

Supplementary figure 7

Distribution of MMPBSA values

Supplementary figure 8

The location of mutations in the core domain of troponin that have been shown to uncouple Ca^{2+} sensitivity from TnI phosphorylation.

TnC is green, TnI is blue and TnT is red
TNNI3 R145G, TNNC1 Y5H, TNNT2 K280N and TNNI3 R21C are HCM linked mutations, TNNC1 G159D and TNNI3 K36Q are DCM linked mutations. Note that deletion of C terminal amino acids of TNNT2 Δ14 and Δ28 also cause HCM and uncouple.

See Messer, A. E. & Marston, S. B. Investigating the role of uncoupling of troponin I phosphorylation from changes in myofibrillar Ca(2+)-sensitivity in the pathogenesis of cardiomyopathy. *Frontiers in physiology* **5**, 315 (2014).

Supplementary table 1

Arpeggio results showing main changes due to phosphorylation

Percentage interactions and deltas for phosphorylation from Arpeggio Significant interactions ordered TnC, TnT and TnI

Supplementary table 2

Cohen's *d* calculated for structural and energetic parameters

Supplementary movies

Movies of models shown in Figure 3

Movies of G159D troponin, unphosphorylated and phosphorylated

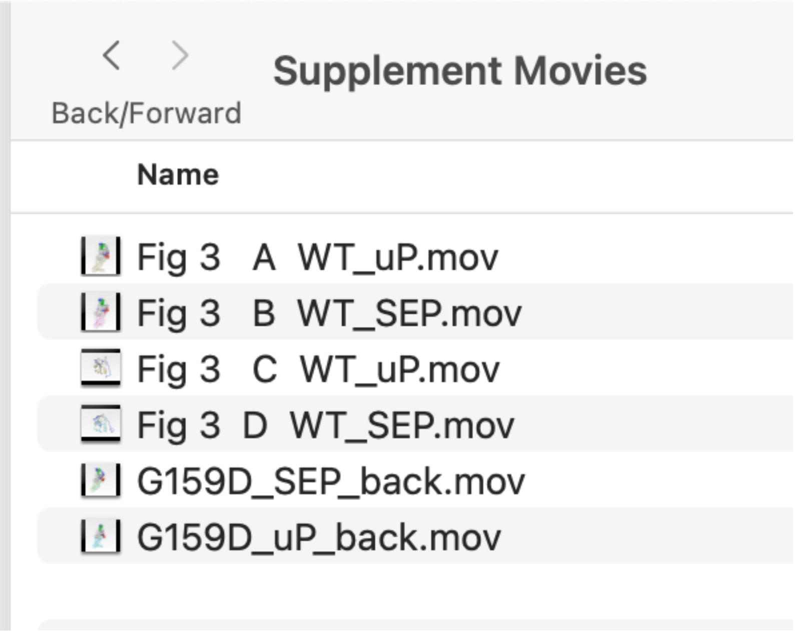

SUPPLEMENTARY DATASETS

Data set 1 **Complete Arpeggio data**

Data set 2 **Pdb files of representative structures referred to in the text**

descriptionof Arpeggio analysis.docx
Arpeggio_aromatic.xlsx
Arpeggio_Hbond.xlsx
Arpeggio_ionic.xlsx
Arpeggio_VdW_clash.xlsx
Arpeggio_VdW_interaction.xlsx
G159D_SEP_run04.pdb
G159D_uP_run02.pdb
WT_SEP_run04.pdb
WT_uP_run01.pdb

RMSF2d

uP SEP uP SEP

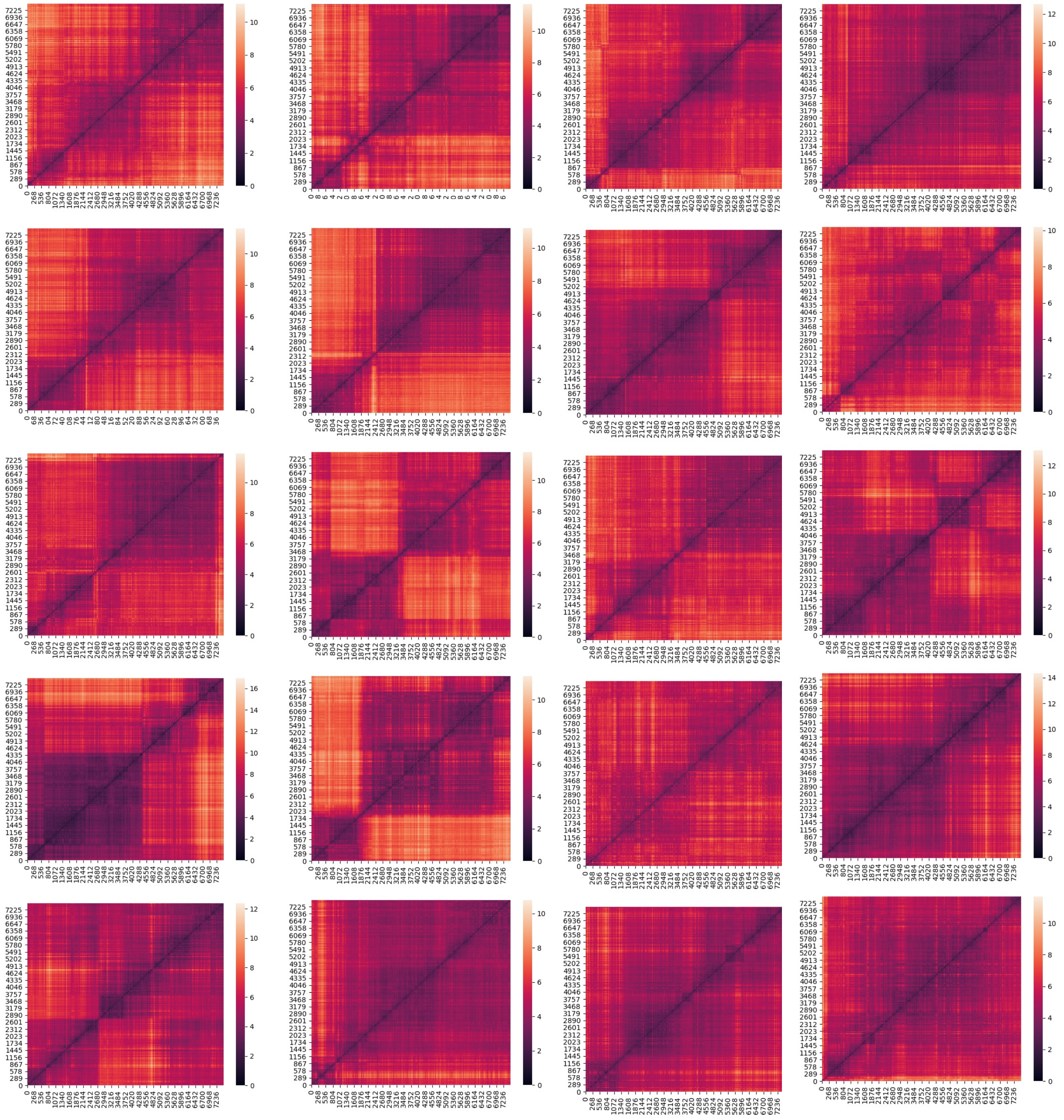

WT G159D

2D RMSD plots of simulation trajectories by comparing frames within each trajectory. No consistent time-dependent large scale conformational changes were observed from the beginning of simulations.
(see Grossfield, A. *et al.* Best Practices for Quantification of Uncertainty and Sampling Quality in Molecular Simulations [Article v1.0]. *Living J Comput Mol Sci* **1**, (2019))

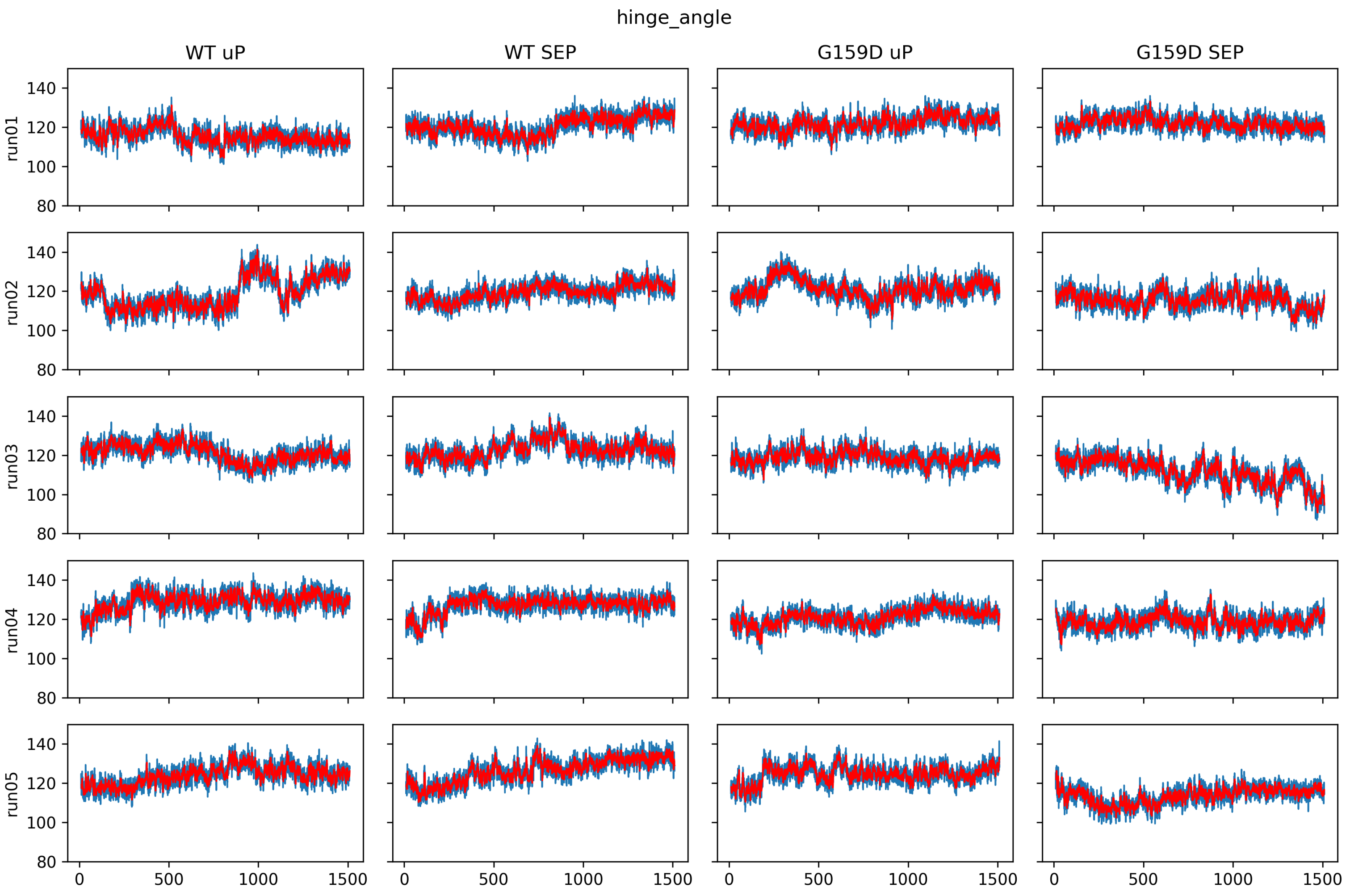

Timelines for the hinge angle parameter for all the 1500ns runs

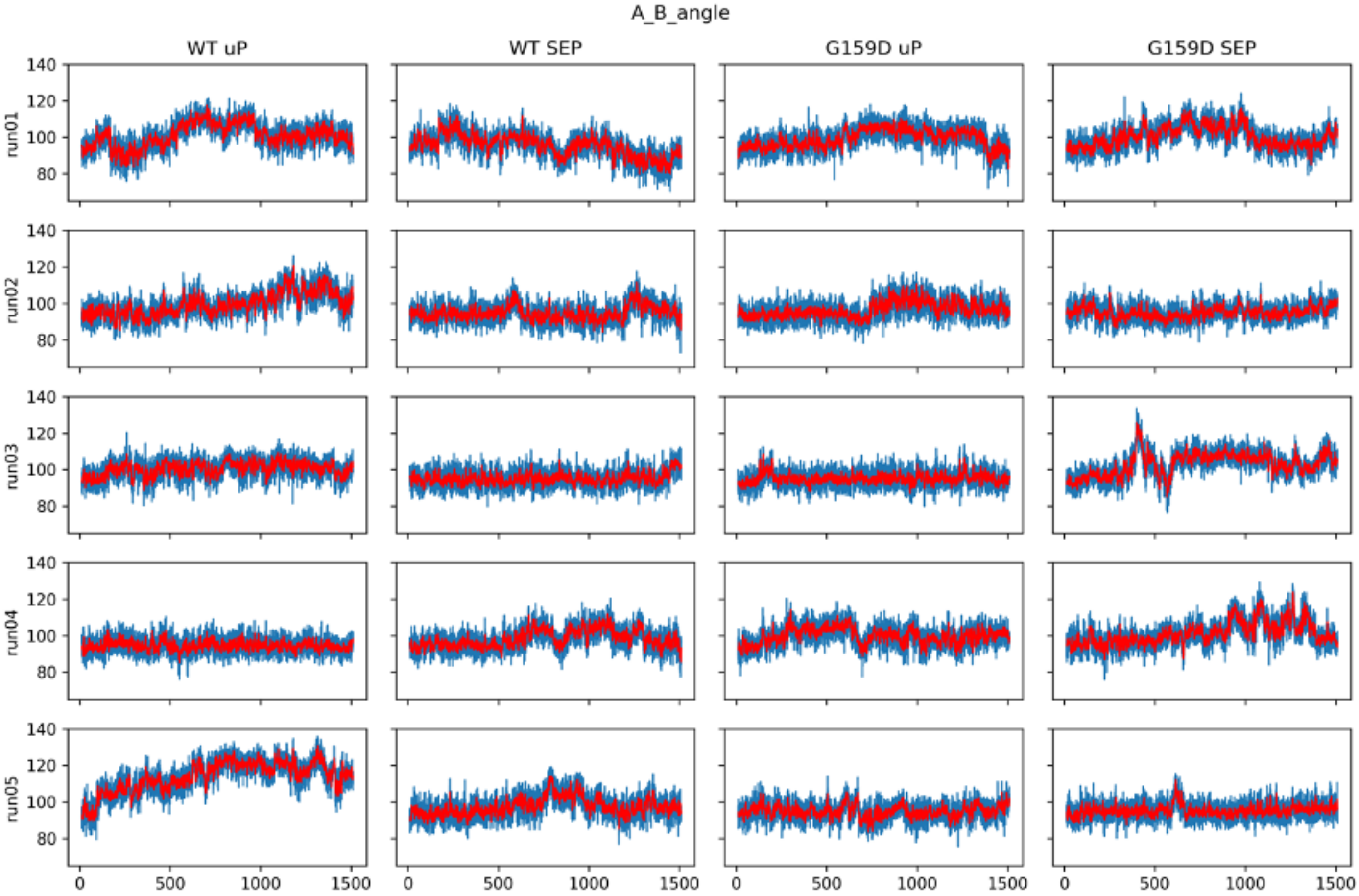

Timelines for the helix A/B angle parameter for all the 1500ns runs

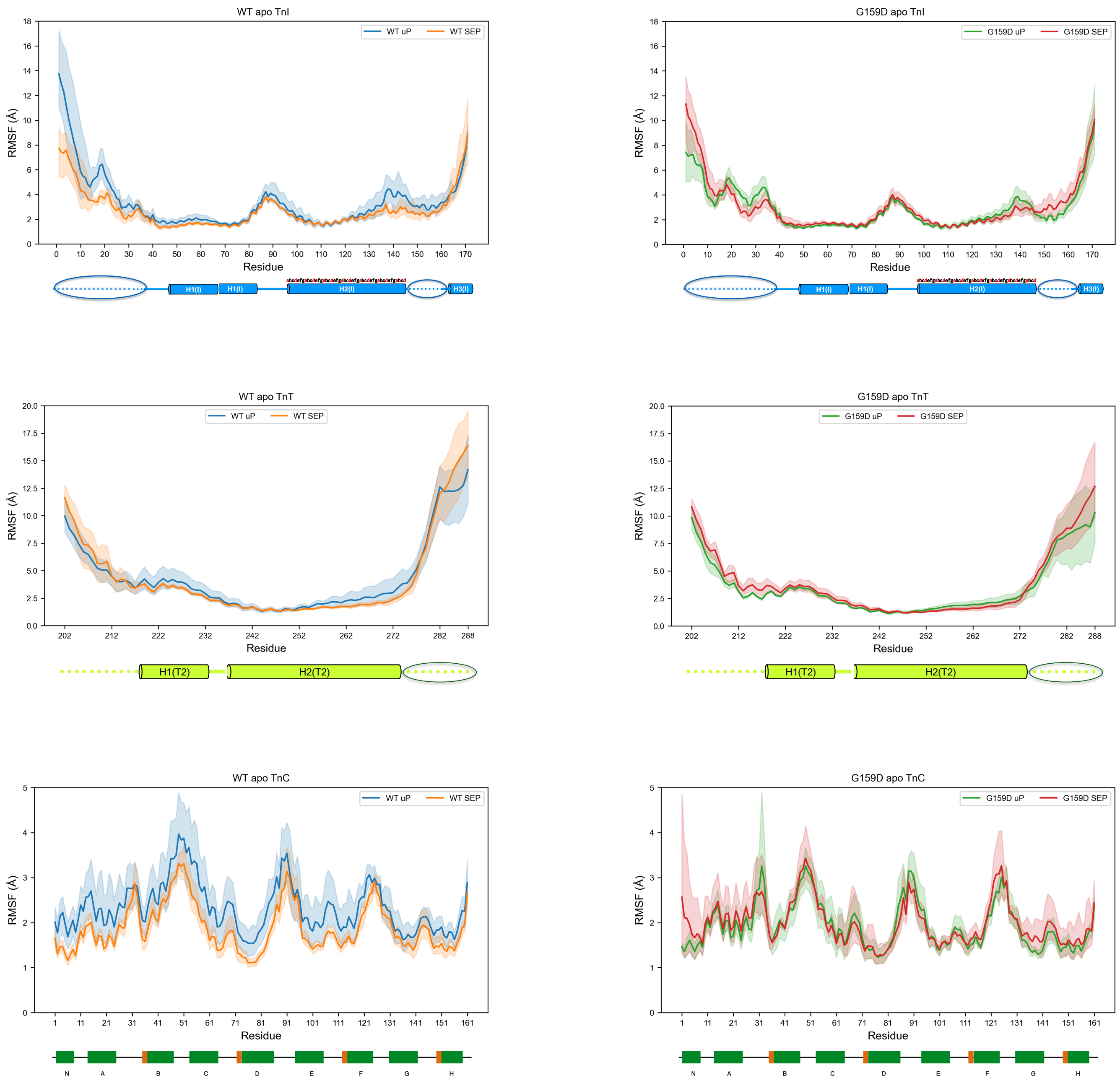

Root Mean Squared Fluctuations (RMSF) of the individual troponin subunits. Wild-type (left) and TnC G159D mutation (right) in unphosphorylated (blue) and phosphorylated (orange) states. The schematics below indicate the structural elements of TnI, TnT and TnC based on Takeda's notation. The disordered regions are ringed. Numerically, the RMSF's are lower with the ff14SB force field compared with previous studies using ff99SB ([redacted]

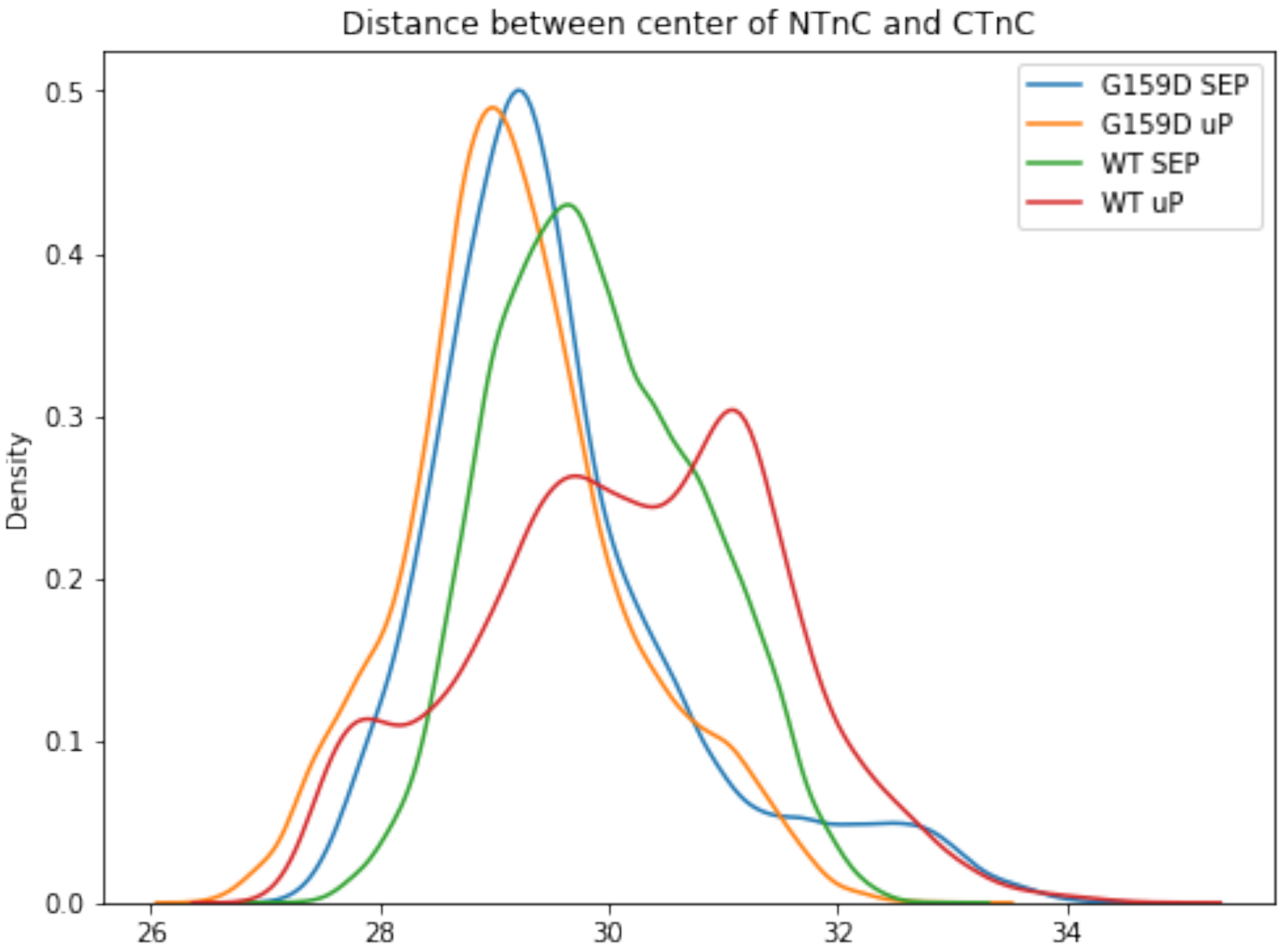

Distribution of length of cTnC 'linking peptide'

Supplementary figure 6

Heat maps of peptide-peptide interactions, calculated from the data in supplementary table 1

A TnI-TnC ionic interactions, uP and P compared

B TnI-TnI ionic interactions, uP and P compared

C TnI-TnC H bond interactions, uP and P compared

D TnT-TnC ionic interactions, uP and P compared

E TnT-TnI ionic interactions, uP and P compared

F TnT-TnI H bond interactions, uP and P compared

G TnC-TnC ionic interactions, uP WT and uP G159D compared

H TnI-TnC G159D ionic interactions, uP and P compared

I TnC-TnC G159D ionic interactions, uP and P compared

S 6A

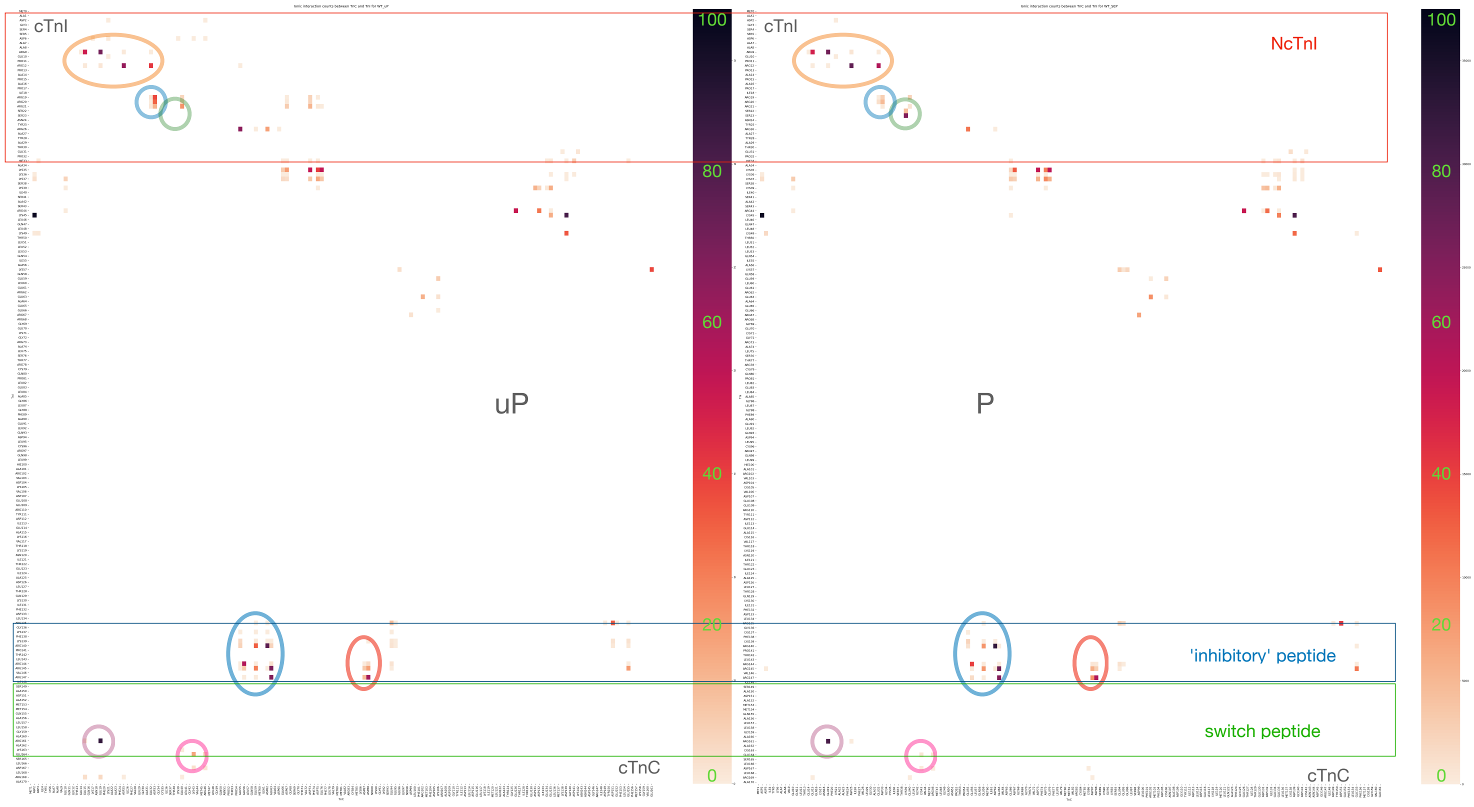

S 6B

The effect of phosphorylation on key intrapeptide ionic interactions of TnI

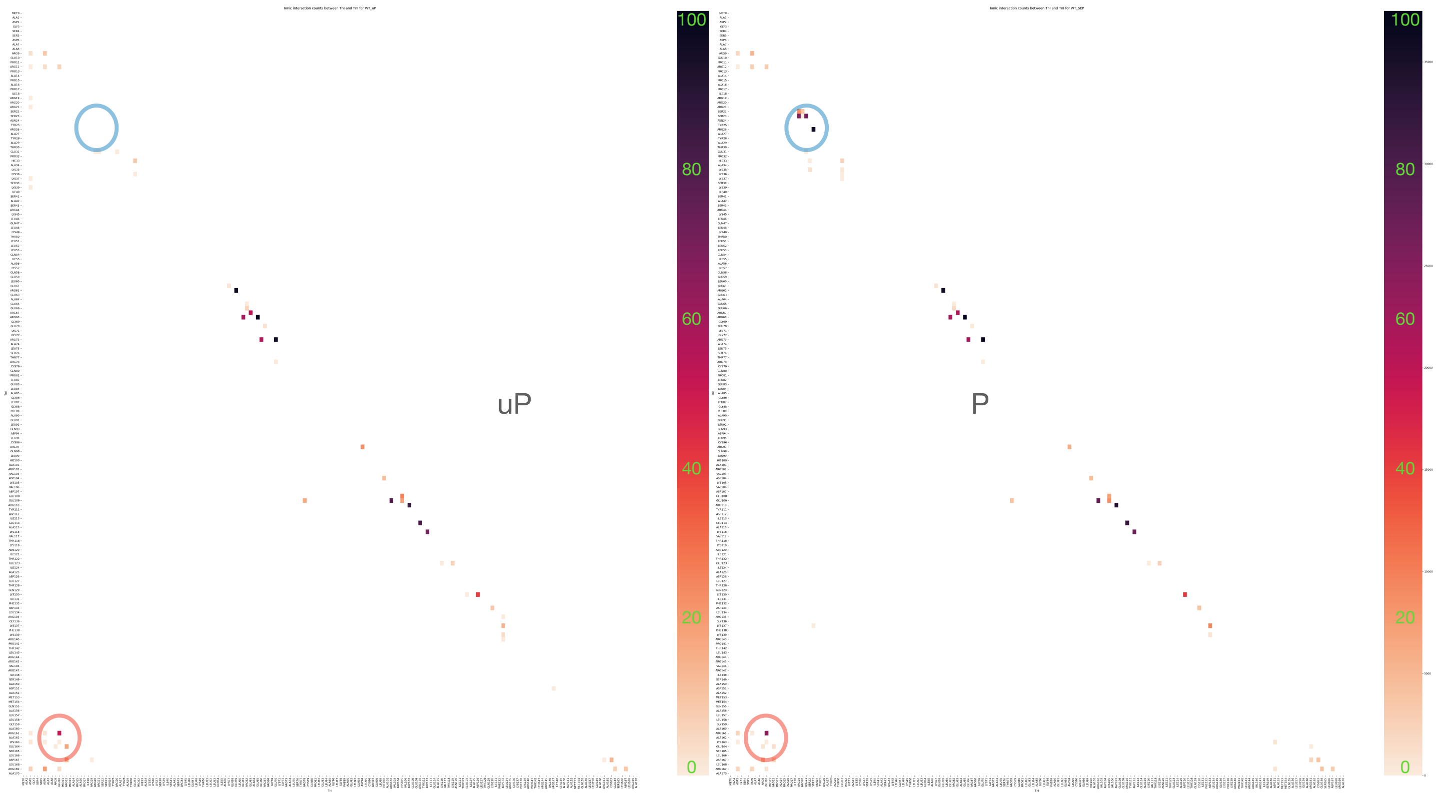

S 6C

The effect of phosphorylation on key H bond interactions between TnI and TnC

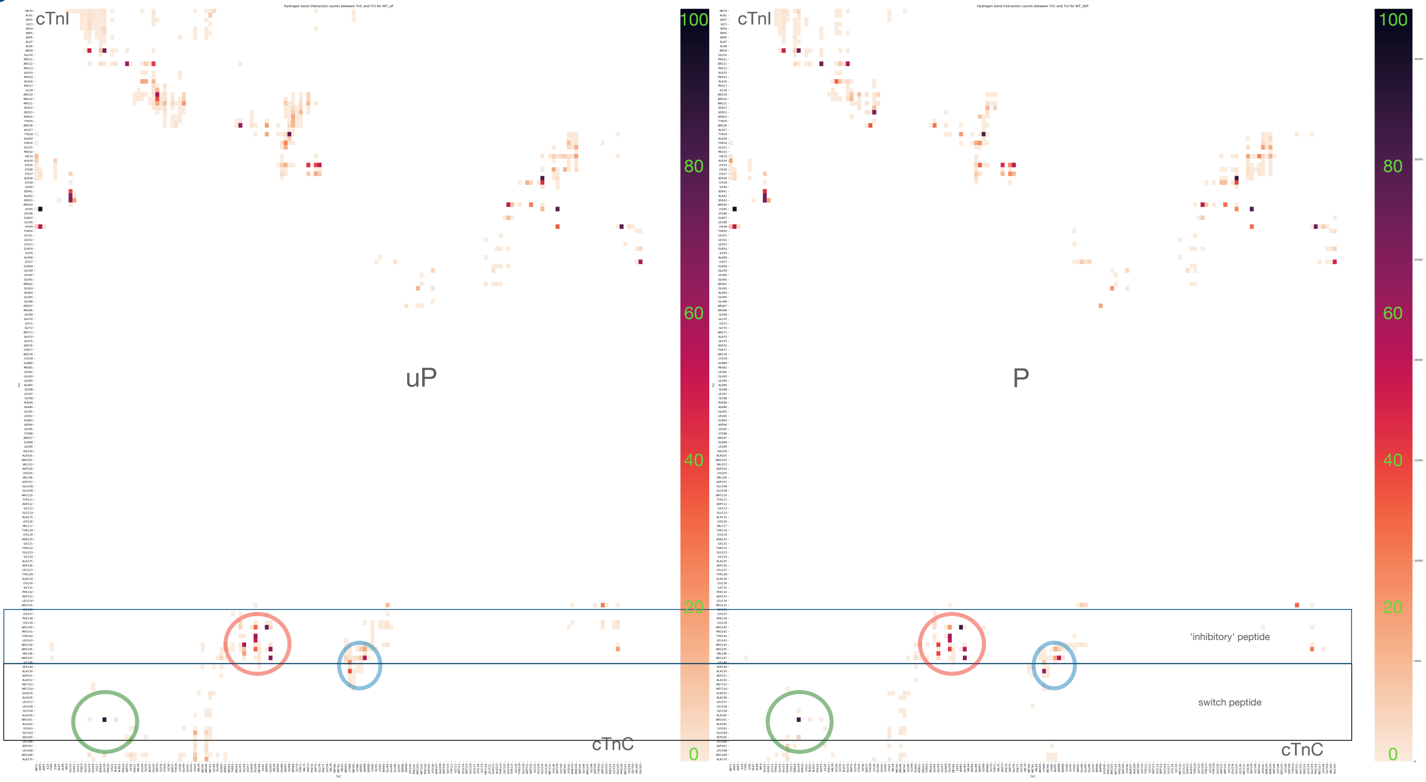

S 6D

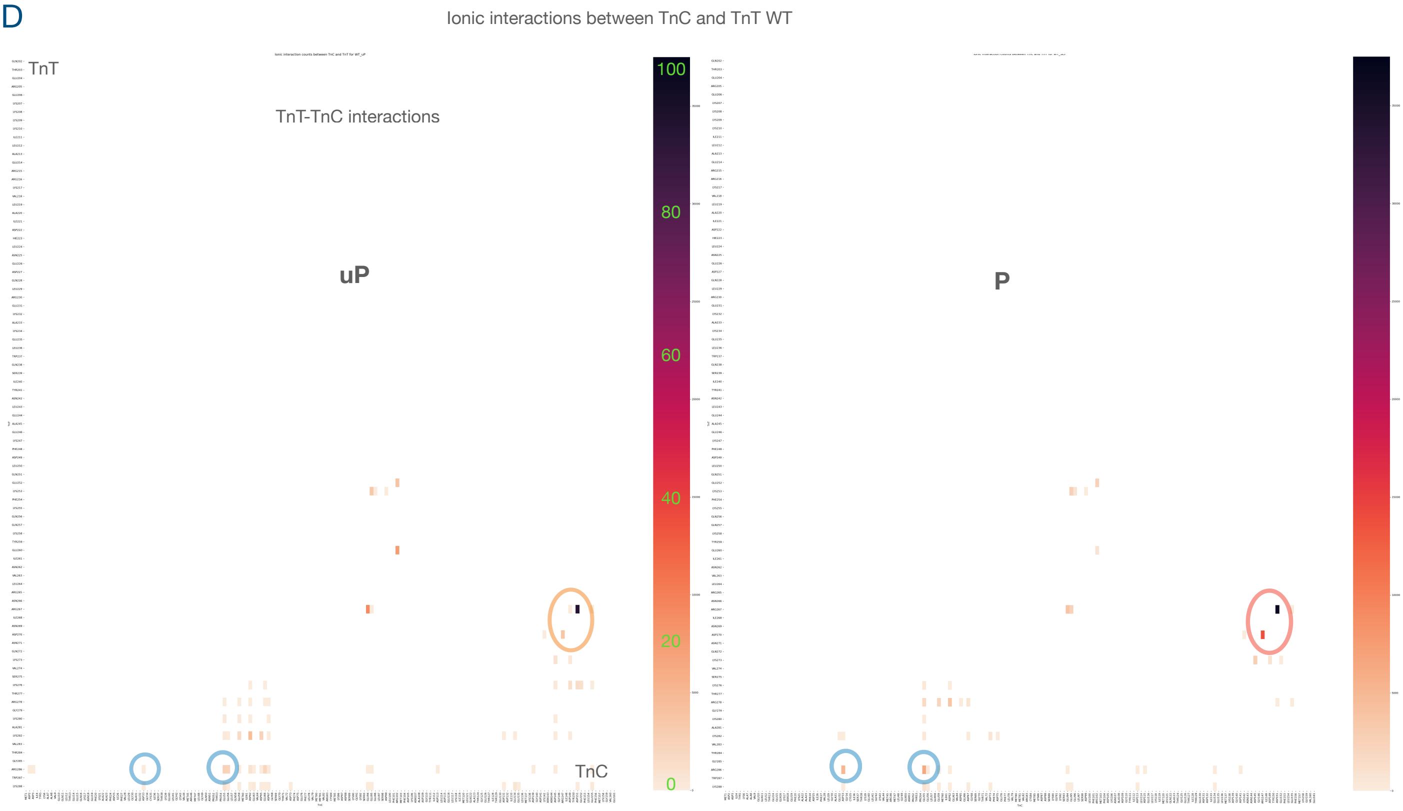
Ionic interactions between TnC and TnT WT
TnT
TnT-TnC interactions
uP
P
TnC
100
80
60
40
20
0

S 6E

Ionic interactions between TnI and TnT WT

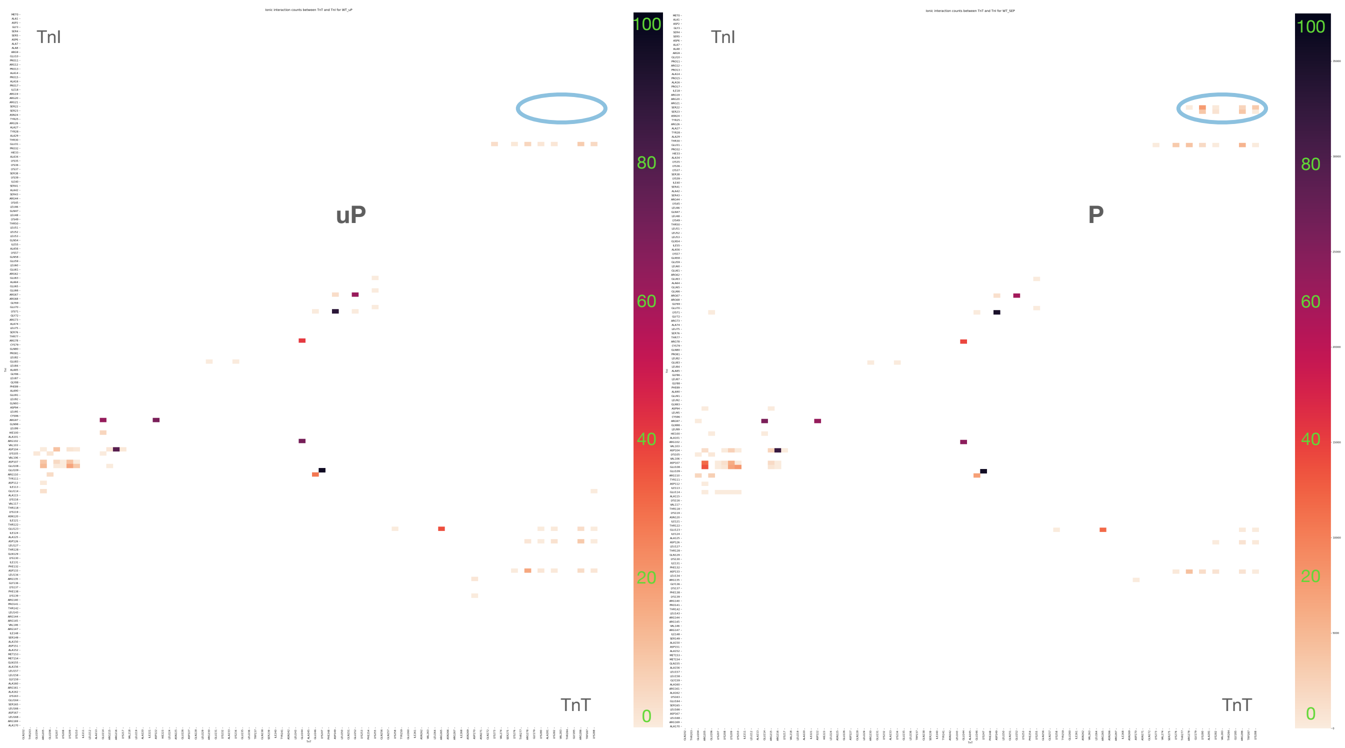

S 6F

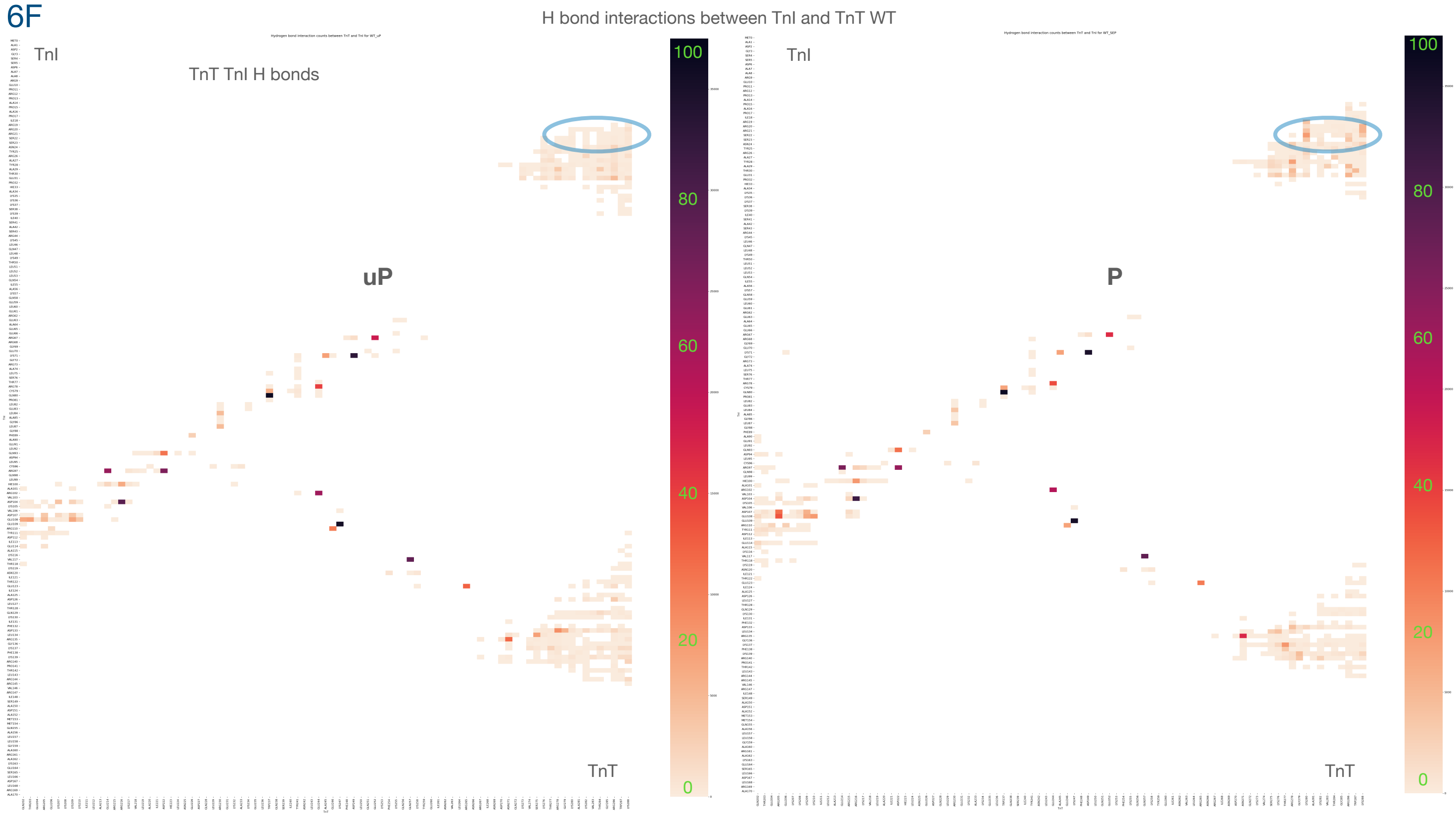
H bond interactions between TnI and TnT WT
TnI
TnT TnI H bonds
uP
TnT
100
80
60
40
20
0
TnI
P
TnT
100
80
60
40
20
0

S6 G

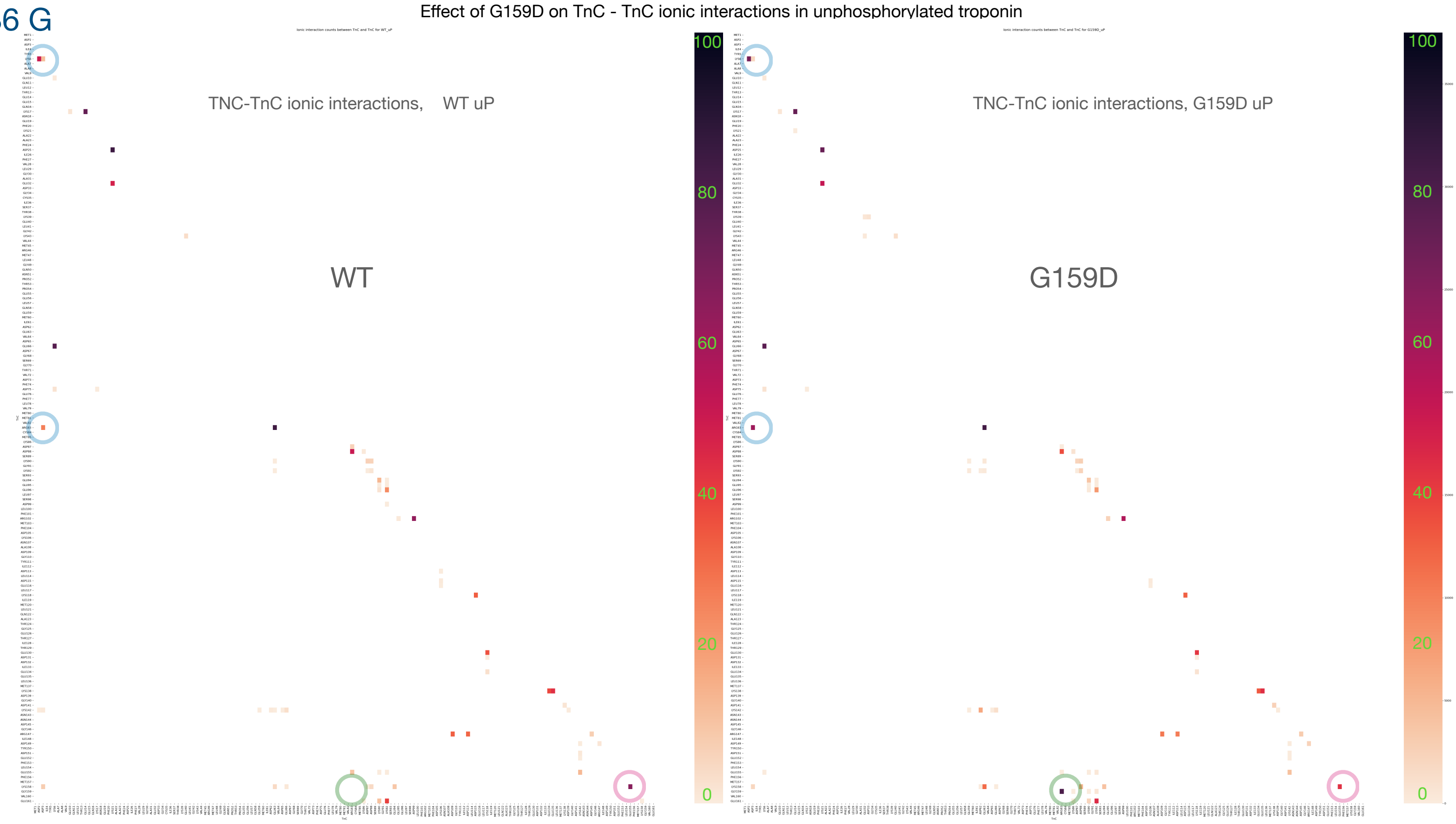
Effect of G159D on TnC - TnC ionic interactions in unphosphorylated troponin
TNC-TnC ionic interactions, WT uP
WT
TNC-TnC ionic interactions, G159D uP
G159D
100
80
60
40
20
0
100
80
60
40
20
0

S 6H

The effect of phosphorylation on key ionic interactions between TnI and TnC in G159D troponin

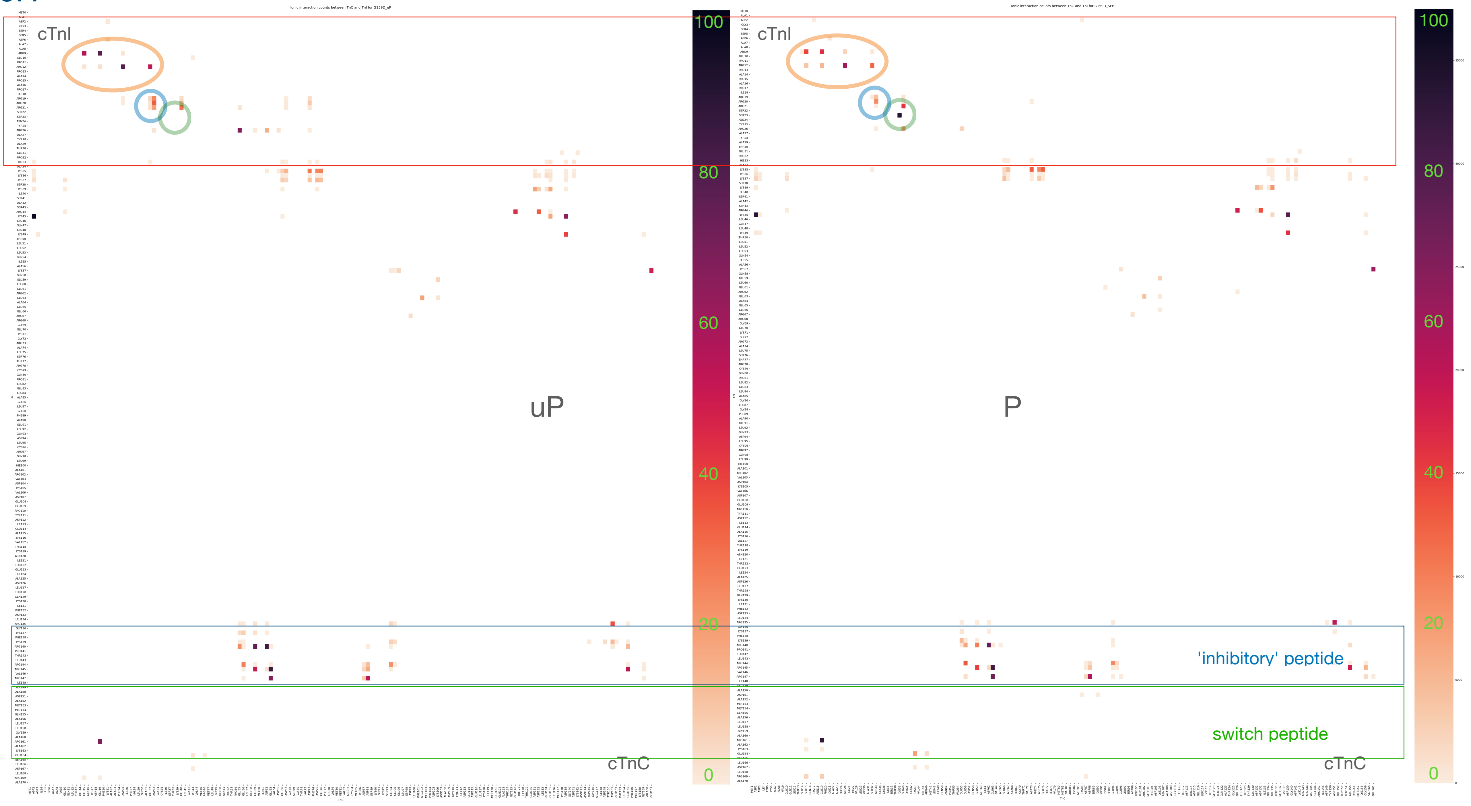

S 6I

The effect of phosphorylation on key intrapeptide interactions of TnC in G159D troponin

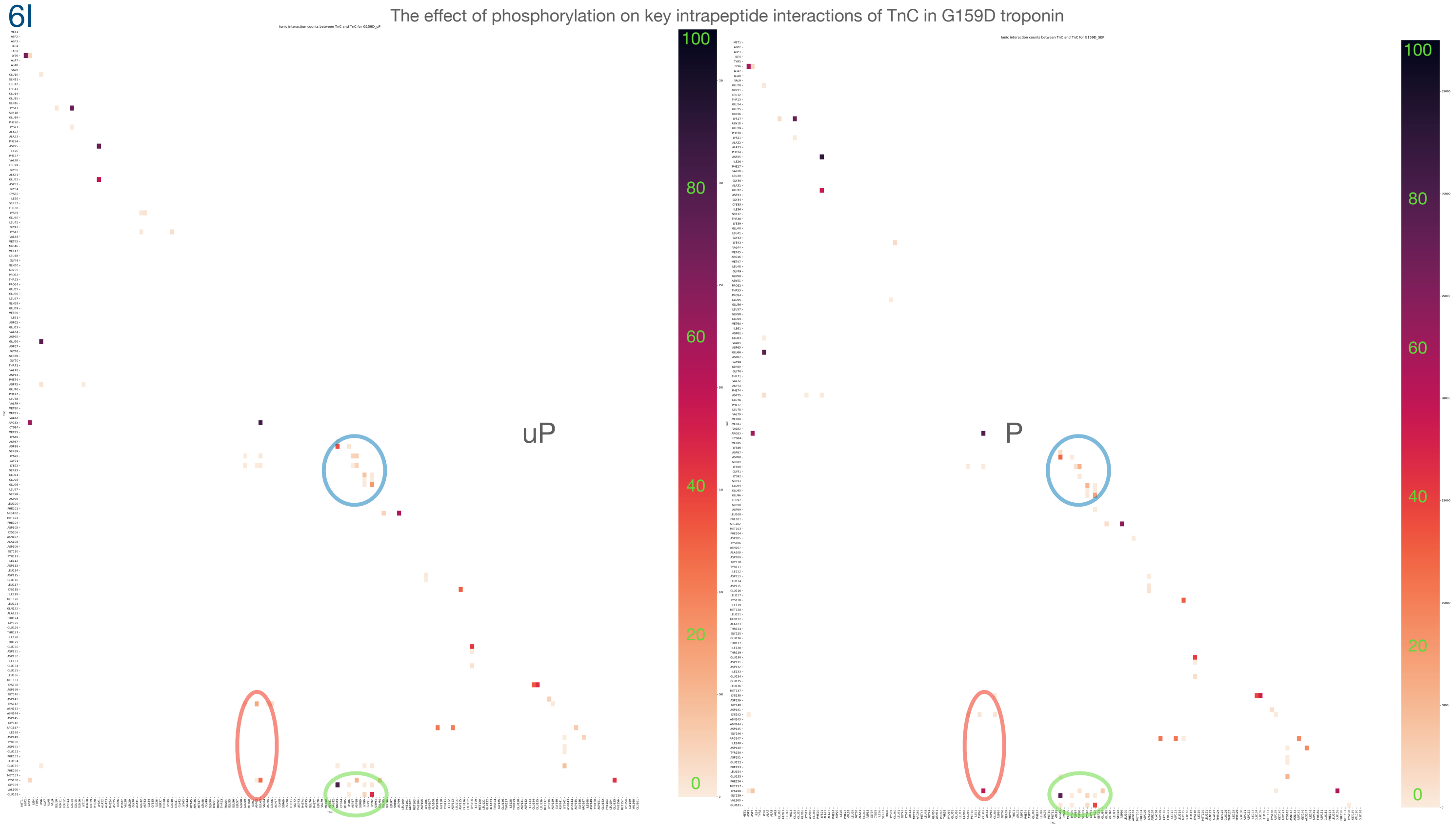

Supplement Figure 7 Distribution of MMPBSA values

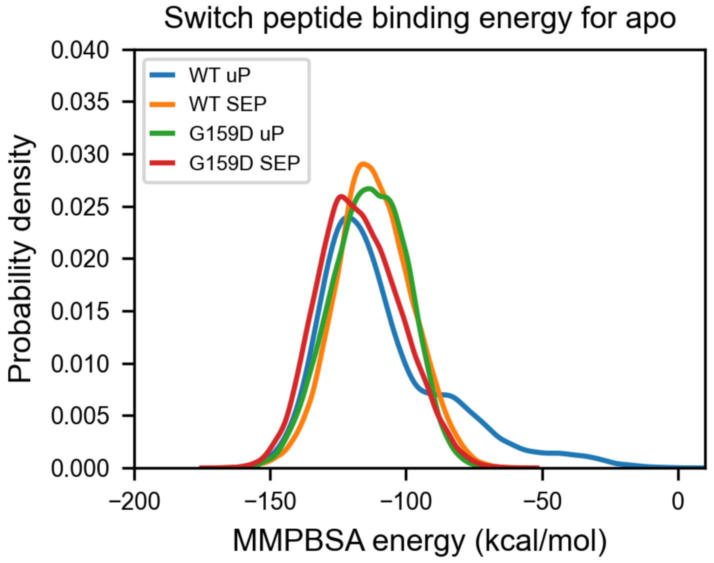

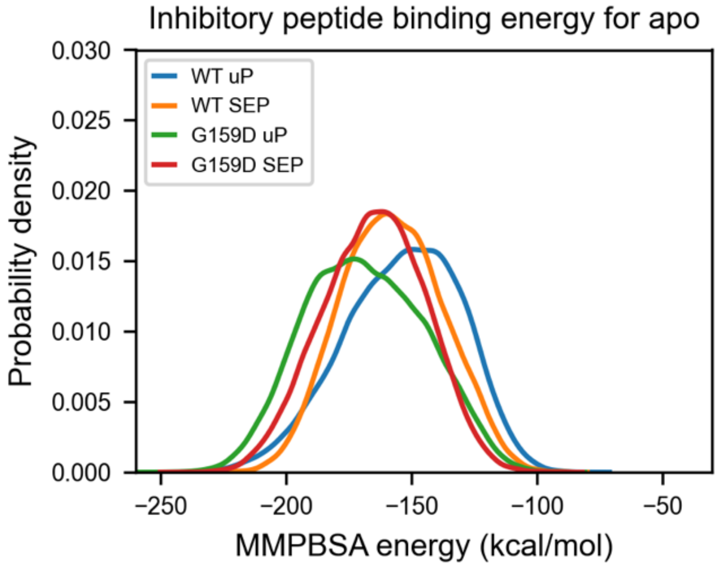

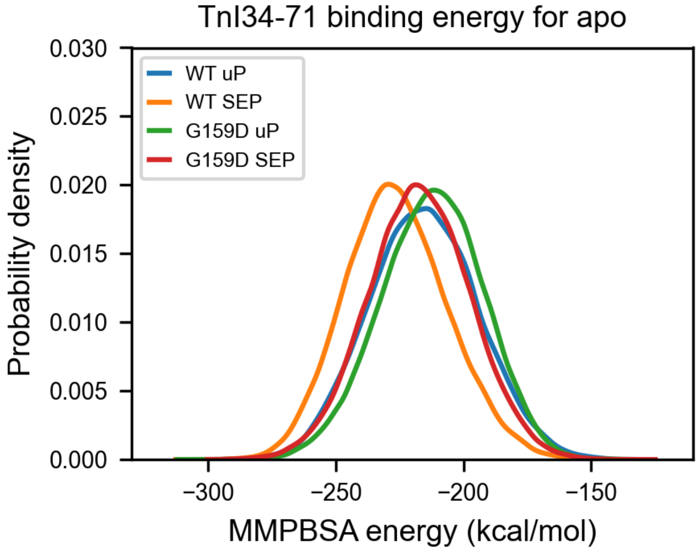

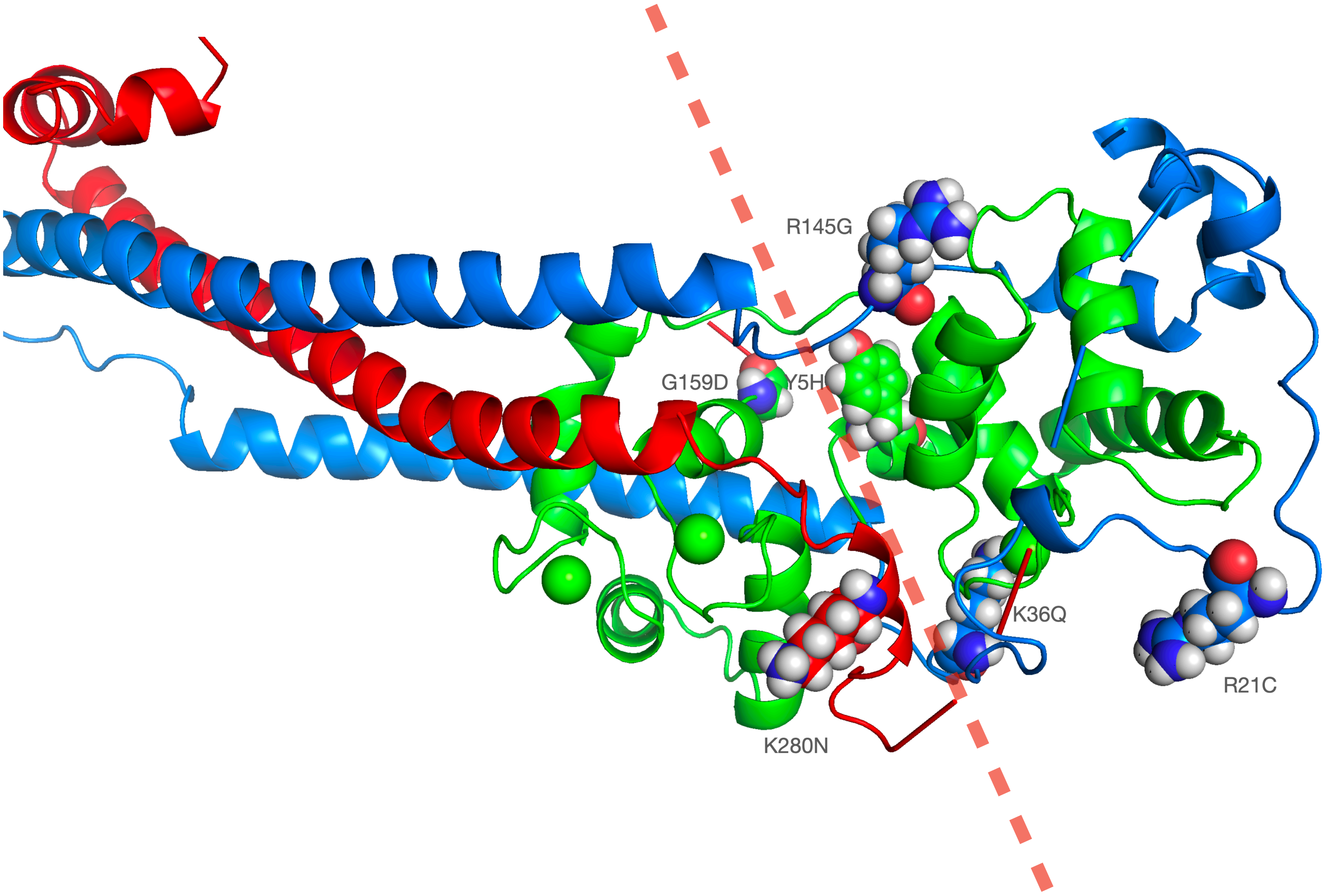

The location of mutations in the core domain of troponin that have been shown to uncouple Ca^{2+} sensitivity from TnI phosphorylation.

TnC is green, TnI is blue and TnT is red

TNNI3 R145G, TNNC1 Y5H, TNNT2 K280N and TNNI3 R21C are HCM linked mutations, TNNC1 G159D and TNNI3 K36Q are DCM linked mutations. Note that deletion of C terminal amino acids of TNNT2 Δ14 and Δ28 also cause HCM and uncouple.

See Messer, A. E. & Marston, S. B. Investigating the role of uncoupling of troponin I phosphorylation from changes in myofibrillar Ca(2+)-sensitivity in the pathogenesis of cardiomyopathy. *Frontiers in physiology* **5**, 315 (2014).

Supplementary Table 1
Percentage interactions and deltas for phosphorylation from Arpeggio
Significant interactions ordered TnC, TnT and TnI

Residue	Residue	Interaction	% WT U/P	% WT Phos	% WT Phos – U/P	%G159D U/P	%G159D Phos	% G159D Phos-U/P
TnC GLU15	TnI ARG9	Ionic	49	48	-1	55	38	-17
TnC GLU19	TnI ARG9	Ionic	70	70	0	76	43	-33
TnC ASP73	TnI LYS35	Ionic	51	52	1	28	34	6
TnC ASP73	TnI LYS37	Ionic	9	14	5	10	0	-10
TnC ASP75	TnI Lys35	Ionic	40	26	-14	25	25	0
TnC ASP75	TnI Lys37	Ionic	17	24	+7	38	33	-5
TnC GLU76	TnI LYS35	Ionic	52	54	2	26	34	8
TnC ASP2	TnI LYS45	Ionic	97	96	-1	97	89	-8
TnC ASP2	TnI Lys49	H-Bond	54	52	-2	77	68	-9
TnC GLU32	TnI ARG12	Ionic	41	56	15	52	32	-20
TnC ASP33	TnI ARG19	Ionic	38	7	-31	25	11	-14
TnC ASP33	TnI ARG20	Ionic	32	12	-20	33	22	-10
TnC ASP33	TnI ARG21	Ionic	14	0	-14	19	0	-19
TnC THR38	TnI ARG26	H-Bond	3	35	32	2	47	45
TnC LYS39	TnI SER22	Ionic	0	12	12	0	0	0
TnC LYS39	TnI SER23	Ionic	0	66	66	0	90	90

TnC GLU40	TnI ARG19	Ionic	5	6	1	4	2	-2
TnC GLU40	TnI ARG20	Ionic	6	0	-6	13	0	-13
TnC GLU40	TnI ARG21	Ionic	22	2	-20	29	42	13
TnC PHE24	TnC PHE74	Aromatic	60	60	0	58	54	-4
TnC PHE24	TnC PHE77	Aromatic	89	93	4	93	93	0
TnC ASP25	TnI ARG12	Ionic	62	74	12	77	59	-18
TnC PHE27	TnC PHE77	Aromatic	22	35	13	27	29	2
TnI ARG19	TnI SER23	Ionic	0	68	68	0	48	48
TnI ARG20	TnI SER22	Ionic	0	10	10	0	40	40
TnI ARG21	TnI SER23	Ionic	0	72	72	0	90	90
TnI SER22	TnI ASN24	H-Bond	27	85	58	33	91	58
TnI SER23	TnI ARG26	Ionic	0	95	95	0	98	98
TnI SER23	TnI TYR25	H-Bond	24	33	9	23	49	26
TnI SER23	TnI ALA27	H-Bond	15	0	-15	31	0	-31
TnC ASP33	TnT ARG286	Ionic	0	13	13	0	8	8
TnC ASP105	TnT TYR259	H-Bond	72	97	25	83	97	14
TnC ALA108	TnT ASN266	H-Bond	11	2	-9	0	18	18
TnC ASP109	TnT ASN262	H-Bond	17	30	13	29	22	-7
TnC ASP109	TnT ASN266	H-Bond	31	57	26	47	40	-7

TNC GLY110	TnT ASN266	H-Bond	25	41	16	27	23	-4
TnC TYR111	TnT ASP270	H-Bond	55	72	17	71	48	-23
TnC ARG147	TnT ASP270	Ionic	9	36	27	6	23	17
TnC ASP151	TnT ARG267	Ionic	90	97	7	76	96	20
TnC ASP151	TnT ASN271	H-Bond	26	28	2	18	6	-12
TnT LYS280	TnI ARG19	H-Bond	0	10	10	0	0	0
TnT LYS280	TnI ARG21	H-Bond	0	10	10	0	0	0
TnT LYS280	TnI SER22	Ionic	0	20	20	0	14	14
TnC GLU19	TnI ARG161	Ionic	86	81	-5	68	87	19
TnC LYS43	TnI GLU164	Ionic	17	1	-16	2	18	16
TnC LYS43	TnI ARG169	H-Bond	8	0	-8	0	0	0
TnC ARG46	TnI ALA170	H-Bond	15	0	-15	0	2	2
TnC CYS84	TnI ALA150	H-Bond	37	59	22	55	50	-5
TnC ARG83	TnC ASP88	Ionic	50	32	-18	37	32	-5
TnC ARG83	TnC GLU155	Ionic	10	0	-10	0	0	0
TnC ARG83	TnC GLY159	Ionic	0	0	0	80	76	-4
TnC ARG83	TnI ARG147	H-Bond	5	0	-5	8	11	3
TnC ASP88	TnC LYS90	H-Bond	23	28	5	17	27	10
TnC ASP88	TnI ARG145	Ionic	18	7	-11	16	10	-6

TnC ASP88	TnI ARG147	Ionic	60	57	-3	52	57	+5
TnC SER89	TnI GLY159	H-Bond	15	27	12	29	15	-14
TnC LYS90	TnC GLU94	Ionic	14	8	-6	11	14	3
TnC LYS90	TnC GLU159	H-Bond	14	11	-3	3	7	4
TnC GLY91	TnC GLY159	H-Bond	32	38	6	29	11	-18
TnC LYS92	TnC GLU161	Ionic	40	31	-9	45	38	-8
TnC SER93	TnC GLU95	H-Bond	63	43	-20	64	62	-2
TnC SER93	TnC GLU96	H-Bond	93	71	-22	89	96	7
TnC SER93	TnC LEU97	H-Bond	77	64	-13	81	86	7
TnC GLU94	TnC SER98	H-Bond	78	65	-13	90	90	0
TnC GLU94	TnC TYR150	H-Bond	42	31	-11	20	18	-2
TnC ASP62	TnI ARG140	Ionic	75	86	11	87	79	-8
TnC GLU63	TnI ARG145	Ionic	67	72	5	85	82	-3
TnC ASP87	TnI ARG145	Ionic	9	15	6	5	0	-5
TnC ASP87	TnI ARG147	Ionic	12	22	10	8	5	-3
TnC GLU56	TnI ARG144	Ionic	56	39	-17	26	35	9
TnC GLU56	TnI ARG145	Ionic	8	0	-8	0	0	0
TnC GLU56	TnI VAL146	H-Bond	19	38	19	21	14	-7
TnC GLU59	TnI ARG140	Ionic	33	21	-12	74	27	-47

TnC GLU59	TnI ARG145	Ionic	26	21	-5	50	40	-10
TnC ASP139	TnI LYS45	Ionic	81	80	-1	64	77	13
TnC ASN143	TnT LYS282	H-Bond	0	0	0	0	14	14
TnC ASN144	TnT LYS282	H-Bond	0	0	0	0	10	10
TnC ARG147	TnT ASP270	Ionic	9	36	27	6	23	17
TnC ASP151	TnT ARG267	Ionic	90	97	7	76	96	20
TnC ASP151	TnT ASN271	H-Bond	26	28	2	18	6	-12
TnC GLU161	TnI LYS57	Ionic	38	35	-3	49	60	11
TnT THR277	TnI LYS137	H-Bond	6	21	15	11	0	-11
TnT ARG278	TnI TYR28	H-Bond	0	18	18	0	13	13
TnT ARG278	TnI ASP133	H-Bond	23	11	-12	40	14	-26
TnT GLY279	TnI ASP133	H-Bond	14	2	-12	12	2	-10
TnT GLY279	TnI LYS139	H-Bond	0	11	11	0	0	0
TnT GLY279	TnI ARG140	H-Bond	0	12	12	0	0	0
TnT LYS280	TnI ARG19	H-Bond	0	10	10	0	0	0
TnT LYS280	TnI ARG21	H-Bond	0	10	10	0	0	0
TnT LYS280	TnI SER22	Ionic	0	20	20	0	14	14
TnT LYS282	TnI GLU31	Ionic	1	0	-1	0	12	12
TnI ARG9	TnI ASP167	Ionic	0	10	10	1	2	1

TnI GLU10	TnI ARG161	Ionic	50	65	15	72	40	-32
TnI GLU10	TnI ARG169	Ionic	3	2	-1	4	20	16
TnI ARG12	TnI GLU164	Ionic	17	3	-14	0	7	7
TnI ARG19	TnI SER22	Ionic	0	23	23	0	42	42
TnI ARG19	TnI SER23	Ionic	0	68	68	0	48	48
TnI ARG20	TnI SER22	Ionic	0	10	10	0	40	40
TnI ARG21	TnI SER23	Ionic	0	72	72	0	90	90
TnI SER22	TnI ASN24	H-Bond	27	85	58	33	91	58
TnI SER23	TnI ARG26	Ionic	0	95	95	0	98	98
TnI SER23	TnI TYR25	H-Bond	24	33	9	23	49	26
TnI SER23	TnI ALA27	H-Bond	15	0	-15	31	0	-31
TnI ASN24	TnI ARG26	H-Bond	47	18	-29	37	17	-20
TnI ASN24	TnI ALA27	H-Bond	62	88	26	60	88	28
TnI ARG26	TnI ARG140	H-Bond	48	71	23	16	62	46
TnI ALA27	TnI ARG140	H-Bond	10	31	21	3	6	3
TnI TYR28	TnI ARG140	H-Bond	7	24	17	6	10	4

Analytical notes for Table 1

WILD-TYPE INTERACTION MODULATED BY PHOSPHORYLATION

NcTnI-NcTnC interactions

Phosphorylation-independent interactions of NcTnI:

NcTnI is anchored, near the N-terminal end by interactions between NcTnI Arg9 and the Glutamines 15 and 19 of NcTnC, which are the start of TnC helix A. . Further phosphorylation-independent interactions anchoring NcTnI to NcTnC are present between the D-helix of TnC Asp73 and NcTnI Lys 35, between Asp 75 and NcTnI Lys 35 and 37 and between Glu76 and Lys 35. Finally NcTnC Asp2 interacts with NcTnI Lys45 and 49 (beginning of the TnI H1 helix) these residues being located on either side of the NcTnC-ITC domain interface.

Phosphorylation-dependent interactions of NcTnI:

The major interaction between NcTnC and *unphosphorylated* NcTnI is TnC Asp33, in the EF hand loop I, with TnI Arg 19,20 and 21 this is significant as Asp 33 is one of the few cardiac specific variants in cTnC (Gly33 in skeletal muscle TnC) (Figure 2C). Upon phosphorylation, there is a cumulative loss of these interactions. This is accompanied by altered NcTnC-NcTnI interactions increasing the NcTnC Asp25 to NcTnI Arg12 interaction and the Glu32 to Arg12, both TnC residues being at the end of the A helix Interactions of Ser 22 and 23 with TnC Lys39 are formed upon phosphorylation.

In the unphosphorylated state there is no ionic interaction of the Ser 22 and 23 with TnC Lys39, upon phosphorylation the interaction with Ser 22 and 23 increases significantly to ~80% (green circle). The resulting shift of NcTnI-NcTnC interactions changes the location of NcTnI on NcTnC and results in Lys39 in the B helix being pulled towards the phosphorylated serines of NcTnI, confirmed by the formation of an interaction between TnC Thr38 and TnI. As a consequence, there is also a very noticeable change in the orientation of the end of the B-Helix relative to the A helix. There is a loss of interaction of NcTnC Glu40 with NcTnI Arg20/21. Uniquely, there is a strong phosphorylation effect on the interaction of the Phenylalanine residues in the A and D helices. Whilst the interaction between the end of the A helix Phe 24 with the start of the D helix Phe 74 and 77 is relatively constant, the very end of the A helix Phe27 increases its interaction upon phosphorylation with the D helix Phe77.

Phosphorylation-dependent interactions of cTnI switch peptide:

The troponin I switch peptide, 149-164, is docked onto the hydrophobic patch of NcTnC formed by helix A and B. It is anchored to NcTnC by a stable interaction between NcTnC Glu19 and cTnI Arg161, but the repositioning of cTnC helix B upon phosphorylation alters the hydrophobic patch structure and results in repositioning of the switch peptide. There is a noticeable loss of interactions between TnC helix B and the C-terminal part of the switch

peptide. The interaction of NcTnC Lys43 with the switch peptide residues Glu164 and Arg169 decreases and the interaction of cTnC Arg 46 with ALA170 also decreases. At the N-terminal end of the switch peptide there is increased hydrogen bonding between cTnC Cys84 and cTnI Ala150 and cTnC Ser89 and Gly91 with cTnI Gly159.

Phosphorylation-dependent interactions of cTnI 'inhibitory' peptide:

Repositioning of the TnI switch peptide relative to cTnC helices A and B on phosphorylation alters the adjacent 'inhibitory peptide', TnI 136-148. The cTnC C-helix moves with the B helix, thus altering its interactions with the middle of the 'inhibitory peptide. The arginine's in this peptide (140,144,145,147,149) form ionic interactions with cTnC, notably at the start of Helix C and the end of Helix D that shift on phosphorylation. cTnC Asp62 and Glu63 increase their interactions with cTnI Arg140 and 145; TnC Asp87 also increases its interaction with cTnI Arg 145 and 147. cTnC Glu56 loses interaction with cTnI Arg144 and 145, however, there is and increased interaction with cTnI Val146. cTnC Glu59 also losses its ionic interaction with cTnI Arg140 and 145. Overall, upon phosphorylation, the inhibitory peptide repositions towards the start of C-helix.

Intra NcTnI interactions

The binary nature of the increase in interactions between residues of TnC and TnI described above are also echoed by new intra-NcTnI interactions formed between the phosphate groups of Ser 22 and 23 with TnI Arg 19,21 and 26. There is a very clear preference for the Ser 23 to interact with TnI Arg 19,21 and 26, from 0% in the unphosphorylated case. Only Arg 19 makes significant contact with phosphorylated Ser 22 and Arg 20 only interacts weakly in the phosphorylated state

Intra cTnC interactions

Rearrangement of TnC helix B on phosphorylation alters helices C and D. On phosphorylation, contact between the end of D-helix Arg83 and residues Asp88 and Glu155 is weakened, with the linker moving away from the D helix. At the same time, the linker region, Asp88 and Gly91, move collectively towards C-TnC, accompanied by changes in the E-Helix. At the start of E-helix Ser93 is moving away from the rest of the helix. This is reflected in the reduction of its interactions with Glu95, Glu96 and Leu97. The overall effect is that the TnC linker peptide lengthens upon phosphorylation increasing the distance between the NcTnC and CcTnC domains

cTnC-CcTnT interactions

There is a medium interaction formed between the NcTnC EF1 hand, Asp 33, and the CcTnT Arg 286, upon phosphorylation which correlates with the repositioning of the cTnC Lys 39 and the phosphorylated serines described previously. Interactions between the EF3 hand of the CcTnC and the CcTnT are significantly modulated by phosphorylation. The interaction between CcTnC Asp 105 and TnT Tyr 259 increases significantly, whilst CcTnC

Asp 109 with CcTnT Asn 262 and Asn 266 also increase. CcTnC residues at the end of the EF3 hand Gly 110 and Tyr 111 also experience significant increases in interactions with CcTnT residues Asn 266 and Asp 270. Further phosphorylation induced changes between CcTnC and CcTnT are also observed in the region of the EF4 hand and helix H. Specifically the interaction between CcTnC Arg 147 and CcTnT Asp 270 increases and that of Asp 151 with Arg 267.

cTnI-CcTnT interactions

There is formation of an interaction between the CcTnT Lys280 and the phosphorylated Ser 22 and 23 which is completely absent in the unphosphorylated state. There is also an increased cumulative interaction between TnI Arg 19,20 and 21 and CcTnT Lys280 upon phosphorylation which like the interaction with the Ser 22 and 23 is absent in the unphosphorylated state

Effect of G159D mutation on interactions

Phosphorylation-independent changes

The most significant change from the WT is the formation of a strong, ~80%, ionic interaction between Arg 83, at the bottom of the D-helix and the Asp159, the end of the H-helix (green circle) -Supp 6G. Arg 83 also forms a strong interaction with the N-helix of cTnC which is phosphorylation dependent in the WT at the expense of the internal interaction between Glu155 and Lys 158 (pink circle). The aromatic interactions between the end of A-helix Phe's 24 and 27 with the Phe's 74 and 77 at the start of the D-helix are phosphorylation independent and broadly of the same magnitude as the WT, though the WT exhibits phosphorylation dependence in respect of the Phe 27 interaction with Phe77.

Phosphorylation-dependent changes

Structural differences accompanying phosphorylation of G159D, can be observed in respect of the interaction of the N-helix of cTnC and the beginning of the H1 helix of the NcTnI, specifically the interaction of Glu 10 and Ser 41 and 43, in WT the interactions increase whilst in the G159D they decrease. These differences are further amplified when examining the differences in the anchoring of the NcTnI to the A-helix of NcTnC, the interactions of cTnC Glu15 and 19 with cTnI Arg 9 in WT are constant, whilst for G159D they decrease significantly. These indicate a large loss of anchoring of the NcTnI, however, there is an accompanying increase in the binding of the CcTnI switch peptide to the TnC Glu19 whilst in the WT this is relatively static.

Addressing the self-interactions of cTnI in the G159D mutant in general it is observed that the changes upon phosphorylation mirror those observed in the WT, though often the magnitude of the change is larger than that observed for the WT. This is observed in

particular for the interaction of residues Arg 19 through Ala 27, for example Arg19 with Ser 22 and 23 in the G159D increases from 0% in both cases to ~45%, compared with an asymmetric 23% and 68 in the WT; though the cumulative change is the same. For the interaction of TnI Arg 20 and Arg 21 with Ser 22 and Ser 23 respectively we observe the largest changes in magnitude for G159D with respect to WT. This clearly illustrates that phosphorylation has a more pronounced effect on the local structure in this region of the complex.

Phosphorylation-dependent changes in G159D that are the reverse of WT

TnC Asp25 and Glu32 with TnI Arg12 the interaction in the G159D decreases from whilst in the WT they increase (orange circle 6H). In the linker region cTnC-cTnC interactions(supp 6I) for residues Lys 90 with Glu 94 (blue circle) and Asp159 (green circle) interactions increase upon phosphorylation in G159D whilst for WT they decrease. WT interactions between Ser93 and Glu 96 and Leu 97 decreased substantially upon phosphorylation, but for G159D these increase; this is also observed in the interaction of Glu 94 with Ser 98 which remains constant at 90% whilst in the WT there is a decrease.

CcTnC Ala 108 interaction with CcTnT Asn266 increases from 0% to ~20% in contrast to WT where this is essentially completely lost upon phosphorylation. Interactions of Asp109 with CcTnT Asn 262 and 266 decrease by the same magnitudebut increase in WT. For CcTnC Gly110 with CcTnT Asn 266 there is a small decrease whilst in the WT there is a large increase. The largest difference between the G159D and the WT is for CcTnC Tyr111 interacting with CcTnT Asp 270: in G159Dthis decreases by ~20% whilst for the WT it increases by ~20% on phosphorylation.

In the EF4 hand of G159D CcTnC Asn 143 and 144 interactions with the CcTnT Lys 282 are formed on phosphorylation where there is a complete absence of interaction in the WT both states. There are also a number of more subtle differences between the interaction of the CcTnT and cTnI in G159D compared to the WT, in particular CcTnT Gly 279 with TnI Lys 139 and 140, in WT there is an increase upon phosphorylation whilst in G159D there is no interaction in either state. A similar pattern is seen for CcTnT Lys 280 with NcTnI Arg 19 and 21, in WT these increase upon phosphorylation, whereas in G159D there is no interaction in either state.

Supplementary Table 2

Cohen's d calculated for structural and energetic parameters

A-B angle

	WT SEP	G159D uP	G159D SEP
**WT uP**	0.74	0.63	0.35
**WT SEP**		-0.16	-0.45
**G159D uP**			-0.31

Hinge angle

	WT SEP	G159D uP	G159D SEP
**WT uP**	-0.25	0.02	0.81
**WT SEP**		0.32	1.18
**G159D uP**			0.97

MMPBSA_switch_peptide

	WT SEP	G159D uP	G159D SEP
**WT uP**	0.18	0.26	0.43
**WT SEP**		0.11	0.35
**G159D uP**			0.25

MMPBSA_inhibitory

	WT SEP	G159D uP	G159D SEP
**WT uP**	0.18	0.63	0.51
**WT SEP**		0.49	0.36
**G159D uP**			-0.16

MMPBSA_TnI34_71

	WT SEP	G159D uP	G159D SEP
**WT uP**	0.51	-0.16	0.08
**WT SEP**		-0.69	-0.45
**G159D uP**			0.24

With d ~ 0.2 called small, d ~ 0.5 medium, d ~ 0.8 large, and d ~ 1.2 very large
