# Supplementary material for: Modulation of Structure and Dynamics of Cardiac Troponin by Phosphorylation and Mutations Revealed by Molecular Dynamics Simulations
Source: J Phys Chem B. 2023 Oct 4;127(41):8736–48. doi: 10.1021/acs.jpcb.3c02337 (PMC10591477; doi:10.1021/acs.jpcb.3c02337)
Supplement: Supplementary file 6 — jp3c02337_si_006.zip [file jp3c02337_si_006.zip › supplement dataset/ descriptionof Arpeggio analysis.docx]

**Atomistic Interaction Profiling**

Arpeggio was used to determine the atomistic interactions between all interacting atom pairs based on atom types and positions, for all available frames ^28^. The result was summarised to the residue level where the specific interaction type between residues either present or not for each frame. The interaction data was counted for all trajectories for each system to produce the interaction counts where the highest possible count is the total number of frames for each system (37500).

Excel files:

The residue level interaction counts were separated by interaction types and provided as excel files. Column A and B are the interacting residue pairs. Column D, E, F, G are the counts for WT uP, WT SEP, G159D uP, G159D SEP, respectively.
