# Supplementary material for: Modulation of Structure and Dynamics of Cardiac Troponin by Phosphorylation and Mutations Revealed by Molecular Dynamics Simulations
Source: J Phys Chem B. 2023 Oct 4;127(41):8736–48. doi: 10.1021/acs.jpcb.3c02337 (PMC10591477; doi:10.1021/acs.jpcb.3c02337)
Supplement: Supplementary file 6 — jp3c02337_si_006.zip [file jp3c02337_si_006.zip › supplement dataset/revised Supplementary material text for.pdf]

Supplementary material for

TITLE: **Modulation of structure and dynamics of cardiac troponin by phosphorylation and mutations revealed by molecular dynamics simulations**

AUTHORS: Zeyu Yang[1], Stephen B. Marston[2] and Ian R. Gould[1,]*

1. 1) Department of Chemistry, Imperial College London, Molecular Sciences Research Hub, Shepherd's Bush, London, W12 0BZ, UK and the Institute of Chemical Biology, Imperial College London, Molecular Sciences Research Hub, Shepherd's Bush, London, W12 0BZ, UK
2. 2) National Heart & Lung Institute, Imperial College London, W12 0NN, UK

Zeyu Yang https://orcid.org/0000-0002-2957-8063 Steve B Marston https://orcid.org/0000-0001-6054-6070 Ian R Gould https://orcid.org/0000-0003-3559-0234

**corresponding author e-mail i.gould@imperial.ac.uk*

Supplementary data FIGURES Supplementary figure 1

2D RMSD plots of simulation trajectories comparing frames within each trajectory. No consistent time-dependent large scale conformational changes were observed from the beginning of simulations.
(see Grossfield, A. *et al.* Best Practices for Quantification of Uncertainty and Sampling Quality in Molecular Simulations [Article v1.0]. *Living J Comput Mol Sci* 1, (2019))

Supplementary Figure 2

Timelines for the hinge angle parameter for all the 1500ns runs

Supplementary Figure 3

Timelines for the helix A/B angle parameter for all the 1500ns runs

Supplementary figure 4

Root Mean Squared Fluctuations (RMSF) of the individual troponin subunits. Wild-type (left) and TnC G159D mutation (right) in unphosphorylated (blue) and phosphorylated (orange) states. The schematics below indicate the structural elements of TnI, TnT and TnC based on Takeda's notation. The disordered regions are ringed. Numerically, the RMSF's are lower with the ff14SB force field compared with previous studies using ff99SB.

Supplementary figure 5

Distribution of length of cTnC 'linking peptide'

Supplementary figure 6

Heat maps of peptide-peptide interactions, calculated from the data in supplementary table 1

1. A TnI-TnC ionic interactions, uP and P compared
2. B TnI-TnI ionic interactions, uP and P compared
3. C TnI-TnC H bond interactions, uP and P compared
4. D TnT-TnC ionic interactions, uP and P compared
5. E TnT-TnI ionic interactions, uP and P compared
6. F TnT-TnI H bond interactions, uP and P compared
7. G TnC-TnC ionic interactions, uP WT and uP G159D compared
8. H TnI-TnC G159D ionic interactions, uP and P compared
9. I TnC-TnC G159D ionic interactions, uP and P compared

Supplementary figure 7

Distribution of MMPBSA values

Supplementary figure 8

The location of mutations in the core domain of troponin that have been shown to uncouple Ca^{2+} sensitivity from TnI phosphorylation.

TnC is green, TnI is blue and TnT is red
TNNI3 R145G, TNNC1 Y5H, TNNT2 K280N and TNNI3 R21C are HCM linked mutations, TNNC1 G159D and TNNI3 K36Q are DCM linked mutations. Note that deletion of C terminal amino acids of TNNT2 Δ14 and Δ28 also cause HCM and uncouple.

See Messer, A. E. & Marston, S. B. Investigating the role of uncoupling of troponin I phosphorylation from changes in myofibrillar Ca(2+)-sensitivity in the pathogenesis of cardiomyopathy. *Frontiers in physiology* **5**, 315 (2014).

Supplementary table 1

Arpeggio results showing main changes due to phosphorylation

Percentage interactions and deltas for phosphorylation from Arpeggio Significant interactions ordered TnC, TnT and TnI

Supplementary table 2

Cohen's *d* calculated for structural and energetic parameters
