# Supplementary material for: Modulation of Structure and Dynamics of Cardiac Troponin by Phosphorylation and Mutations Revealed by Molecular Dynamics Simulations
Source: J Phys Chem B. 2023 Oct 4;127(41):8736–48. doi: 10.1021/acs.jpcb.3c02337 (PMC10591477; doi:10.1021/acs.jpcb.3c02337)

**Supplementary Table 2**

Cohen’s *d*

A-B angle

|  | WT SEP | G159D uP | G159D SEP |
| --- | --- | --- | --- |
| WT uP | 0.74 | 0.63 | 0.35 |
| WT SEP |  | -0.16 | -0.45 |
| G159D uP |  |  | -0.31 |

Hinge angle

|  | WT SEP | G159D uP | G159D SEP |
| --- | --- | --- | --- |
| WT uP | -0.25 | 0.02 | 0.81 |
| WT SEP |  | 0.32 | 1.18 |
| G159D uP |  |  | 0.97 |

MMPBSA_switch_peptide

|  | WT SEP | G159D uP | G159D SEP |
| --- | --- | --- | --- |
| WT uP | 0.18 | 0.26 | 0.43 |
| WT SEP |  | 0.11 | 0.35 |
| G159D uP |  |  | 0.25 |

MMPBSA_inhibitory

|  | WT SEP | G159D uP | G159D SEP |
| --- | --- | --- | --- |
| WT uP | 0.18 | 0.63 | 0.51 |
| WT SEP |  | 0.49 | 0.36 |
| G159D uP |  |  | -0.16 |

MMPBSA_TnI34_71

|  | WT SEP | G159D uP | G159D SEP |
| --- | --- | --- | --- |
| WT uP | 0.51 | -0.16 | 0.08 |
| WT SEP |  | -0.69 | -0.45 |
| G159D uP |  |  | 0.24 |

With d ~ 0.2 called small, d ~ 0.5 medium, d ~ 0.8 large, and d ~ 1.2 very large

**Supplement Figure 7**


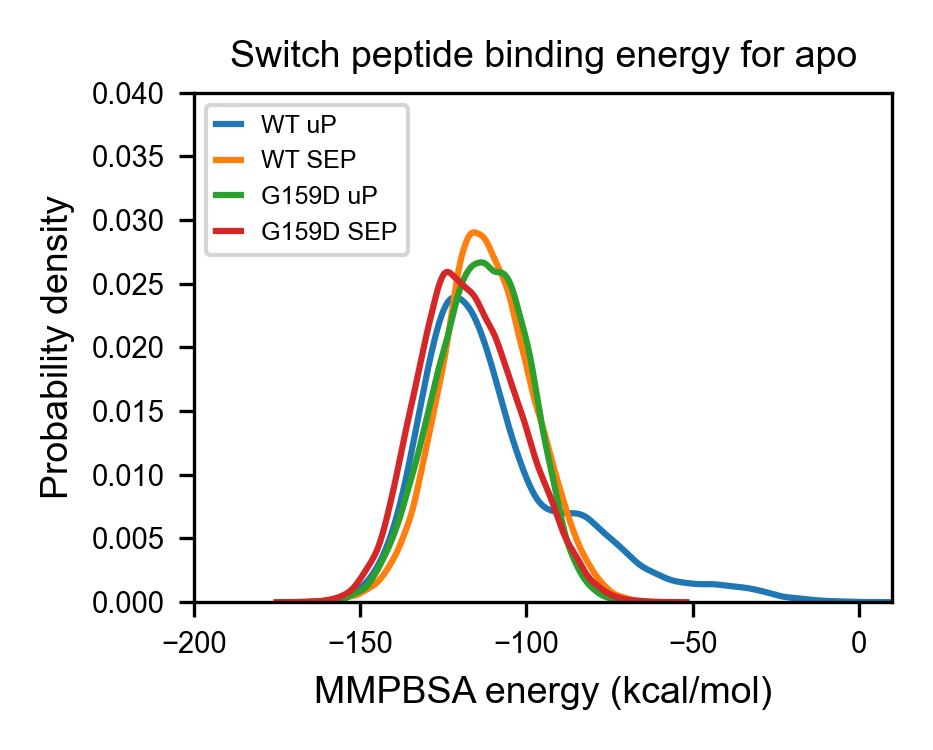

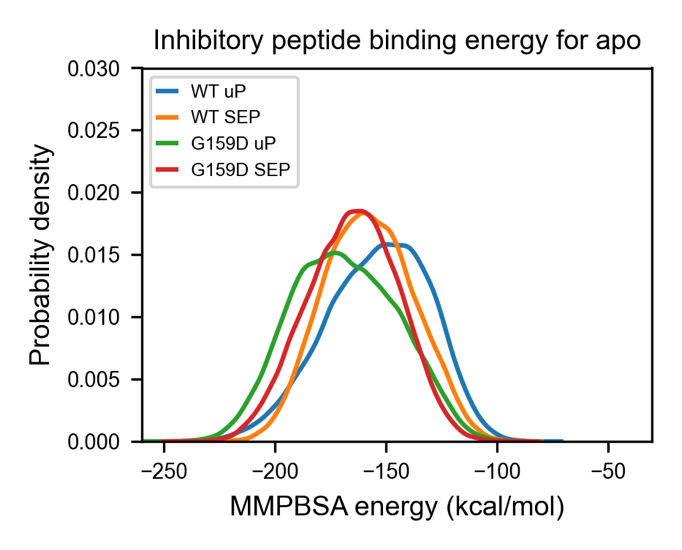


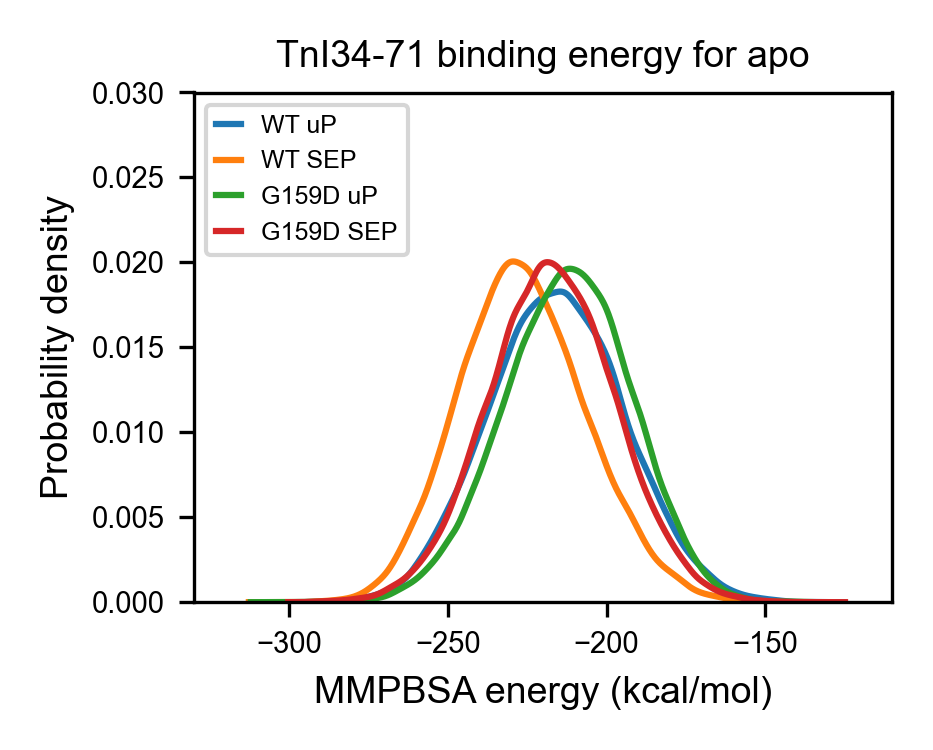

Supplement: Supplementary file 6 — jp3c02337_si_006.zip [file jp3c02337_si_006.zip › supplement dataset/MMPBSA plots, effect size.docx]
