# Supplementary material for: Modulation of Structure and Dynamics of Cardiac Troponin by Phosphorylation and Mutations Revealed by Molecular Dynamics Simulations
Source: J Phys Chem B. 2023 Oct 4;127(41):8736–48. doi: 10.1021/acs.jpcb.3c02337 (PMC10591477; doi:10.1021/acs.jpcb.3c02337)
Supplement: Supplementary file 6 — jp3c02337_si_006.zip [file jp3c02337_si_006.zip › supplement dataset/Supp table 2.pdf]

Supplementary Table 2

Cohen's *d*

A-B angle

	WT SEP	G159D uP	G159D SEP
**WT uP**	0.74	0.63	0.35
**WT SEP**		-0.16	-0.45
**G159D uP**			-0.31

Hinge angle

	WT SEP	G159D uP	G159D SEP
**WT uP**	-0.25	0.02	0.81
**WT SEP**		0.32	1.18
**G159D uP**			0.97

MMPBSA_switch_peptide

	WT SEP	G159D uP	G159D SEP
**WT uP**	0.18	0.26	0.43
**WT SEP**		0.11	0.35
**G159D uP**			0.25

MMPBSA_inhibitory

	WT SEP	G159D uP	G159D SEP
**WT uP**	0.18	0.63	0.51
**WT SEP**		0.49	0.36
**G159D uP**			-0.16

MMPBSA_TnI34_71

	WT SEP	G159D uP	G159D SEP
**WT uP**	0.51	-0.16	0.08
**WT SEP**		-0.69	-0.45
**G159D uP**			0.24

With d ~ 0.2 called small, d ~ 0.5 medium, d ~ 0.8 large, and d ~ 1.2 very large
