# Supplementary figures and images for: Modulation of Structure and Dynamics of Cardiac Troponin by Phosphorylation and Mutations Revealed by Molecular Dynamics Simulations
Source: J Phys Chem B. 2023 Oct 4;127(41):8736–48. doi: 10.1021/acs.jpcb.3c02337 (PMC10591477; doi:10.1021/acs.jpcb.3c02337)

## Supplement Figure 7

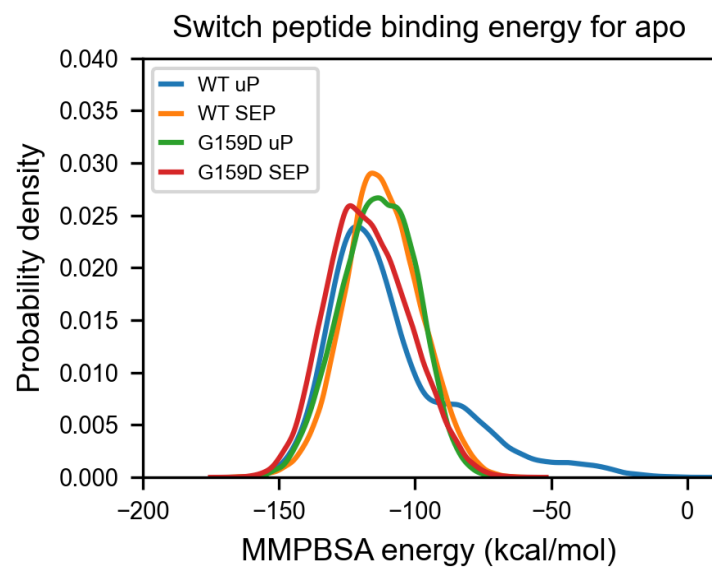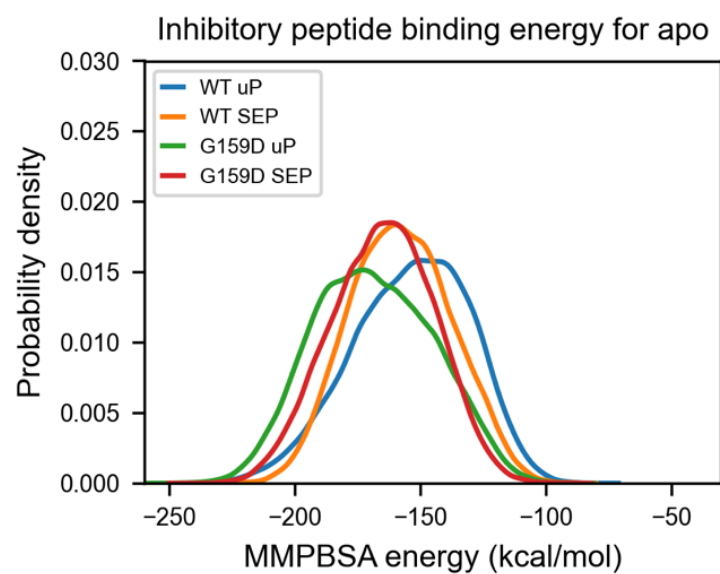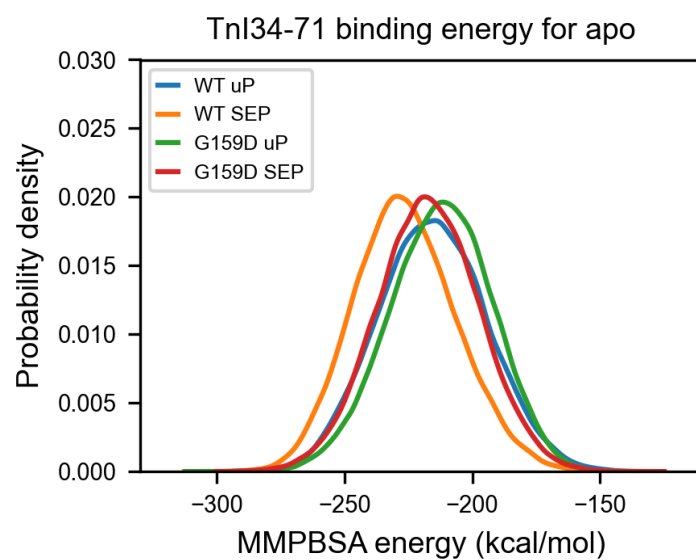

Supplement: Supplementary file 6 — jp3c02337_si_006.zip [file jp3c02337_si_006.zip › supplement dataset/MMPBSA plots,supp7.pdf]

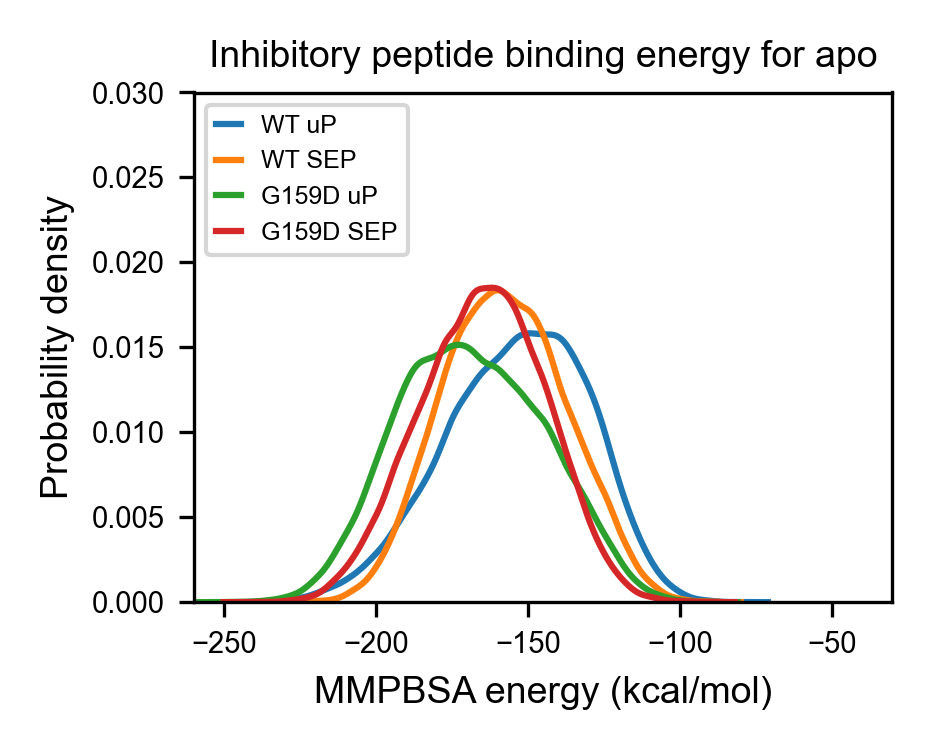

Supplement: Supplementary file 6 — jp3c02337_si_006.zip [file jp3c02337_si_006.zip › supplement dataset/apo.png]
